# Cornin protects against cerebral ischemia/reperfusion injury by preventing autophagy via the PI3K/Akt/mTOR pathway

**DOI:** 10.1186/s40360-022-00620-3

**Published:** 2022-10-24

**Authors:** Tianchi Lan, Yangyang Xu, Shucui Li, Ning Li, Shuping Zhang, Haibo Zhu

**Affiliations:** 1 Department of Pharmacology, Binzhou Medical University, Yantai, Shandong 264003 People’s Republic of China; 2 Department of Pharmacy, Binzhou Medical University Hospital, Binzhou, Shandong 256603 People’s Republic of China; 3 School of Public Health and Management, Binzhou Medical University, Yantai, Shandong 264003 People’s Republic of China

**Keywords:** Cornin, Autophagy, Oxygen glucose deprivation/reperfusion, Cerebral ischemia–reperfusion

## Abstract

**Background:**

Ischemia stroke is the leading cause of disability, which is a consequence of vascular occlusion. The purpose of this study is to investigate the effect of cornin which is isolated from the fruit of Verbena officinalis L, against astrocytes autophagy induced by cerebral ischemia/reperfusion (CI/R) injury *in vitro* and *in vivo* and its potential mechanism.

**Methods:**

Cornin at dose of 2.5, 5 and 10 mg/kg were intravenously injected to MCAO rats at 15 min after reperfusion. The infarction volume, blood–brain barrier (BBB), neurological severity score (mNSS), and autophagy related protein were used to evaluated the protective effects and potential mechanism of cornin in autophagy with or without phosphoinositide-3 kinase (PI3K)inhibitor LY294002 and mammalian target of rapamycin (mTOR) small interfering RNA (siRNA) at 24 h after CI/R injury. The potential protective effects and mechanism of cornin at concention of 10 ~ 1000 nM were also evaluated in oxygen glucose deprivation/reperfusion (OGD/R) in U87 cells.

**Results:**

The results suggest that cornin at dose of 5 or 10 mg/kg significantly reduce the cerebral infarction volume and blood–brain barrier (BBB) leakage, and improve neurological recovery in MCAO rats. Cleaved caspase-3 and Bax levels were significantly decreased, while B-cell lymphoma-2 (Bcl-2) and the apoptosis regulator ratio (Bcl-2/Bax) were markedly increased when treated with 2.5–10 mg/kg cornin. The obvious decreased expressions of glial fibrillary acidic protein (GFAP), myosin-like BCL2 interacting protein (Beclin-1) and microtubule-associated protein light chain 3 II (LC3-II) and increased of neuronal nuclei (NeuN), sequestosome-1 (p62), phosphorylated mTOR (p-mTOR), and phosphorylated Akt (p-Akt) were observed in MCAO rats treated with 10 mg/kg cornin, which was counteracted by LY294002. The expression of autophagy-related proteins with or without LY294002 and mTOR siRNA presented the similar results as *in vitro* in OGD/R in U87 cells.

**Conclusions:**

These results indicate that cornin improved neurological recovery after cerebral ischemia injury by preventing astrocytes autophagy induced by CI/R via the PI3K/Akt/mTOR signaling pathway.

**Supplementary Information:**

The online version contains supplementary material available at 10.1186/s40360-022-00620-3.

## Introduction

Stroke is an acute cerebrovascular disease that has high rates of morbidity, disability, and mortality [1]. Ischemic stroke is present 80%-85% of all cases of stroke and may serious cause damage due to reperfusion after stroke. Currently, the most effective drug treatment for ischemia stroke is to give intravenous tissue plasminogen activator (tPA). However, the administration time window within 4.5 h of stroke symptom onset severely limits the scope of tPA use [2]. Neuroprotectants are potential treatments for ischemic stroke. Although no neuroprotectants have been approved for market, several neuroprotectants based on different mechanisms under preclinical or clinical development [3]. These neuroprotectants are very likely to be approved for marketing and used for the treatment of ischemic stroke in future.

Autophagy plays an important role in cerebral ischemic injury, in which causes progressive brain degeneration in animal and cellular models [4–6]. Autophagy activation occurs under special circumstances, particularly after the application of strong external stimuli such as intracellular damage due to hypoxia and ischemia, starvation, or viral infection. However, over activation of autophagy may result in cell death, which leads to autophagy-mediated or programmed cell death [7, 8]. Microtubule-associated protein light chain 3 II (LC3-II) and myosin-like BCL2 interacting protein (Beclin-1) levels were upregulated, whereas sequestosome-1 (p62) was downregulated following cerebral ischemia, consistent with an activation of autophagy [9]. Inhibiting autophagy activation can reduce cerebral ischemia injury and improve cell survival [9]. The atypical serine/threonine protein kinase mammalian target protein of rapamycin (mTOR) controls cell growth and proliferation [10], and is playing an important role in autophagy regulation. phosphatidylinositol 3-kinase (PI3K)/protein kinase B (Akt), a classical autophagy signaling pathway and the upstream signal of mTOR, plays an important regulatory role in mTOR activation [11]. Previous investigations have shown that mTOR-mediated signaling pathway PI3K/RAC-α serine/Akt/mTOR controls autophagy in cerebral ischemia [12]. Furthermore, the activation of mTOR has a negative regulatory effect on autophagy. During the early phase of cerebral ischemia, injury stimulates the differentiation of astrocytes, which protect the survival of neurons and maintain the normal function of the central nervous system. Astrocytes play an important role in neuroregulation and synaptic transmission [13], and human U87 astrocytes were employed as a cellular model in this study [14]. Therefore, astrocytes involved in autophagy through the PI3K/Akt/mTOR signaling pathway may be a potential treatment for ischemic stroke.

Cornin is an iridoid glycoside derived from the fruit of *Verbena officinalis L*. Previous studies confirmed that cornin has inhibition effects in lipid peroxidation [15], cardioprotection against myocardial ischemic injury [16], and brain protection against cerebral ischemic injury [17, 18]. However, the underlying molecular mechanism of brain protective effect during cerebral ischemia and reperfusion is unclear. The aim of the present study was to determine whether cornin improved neurological recovery after cerebral ischemia injury by prevented astrocytes autophagy induced by cerebral ischemia/reperfusion (CI/R), as well as the potential mechanism signaling, and if so, to determine its underlying neuroprotective mechanism using U87 cells and CI/R rat model.

## Materials and methods

### Drugs and reagents

Cornin (purity > 99.0%, molecular formula: C_17_H_24_O_10_, molecular weight: 388.37) was obtained from SenBeiJia Biological Technology Co., Ltd. (Nanjing, China).

Antibodies against mTOR (Lot No: 2972S, 10), phospho-mTOR (Lot No: 2971S, 26), Akt (Lot No: 9272S, 28), phospho-Akt (Lot No: 13038S, 5), Bax (Lot No: 14796S, 2), and Bcl-2 (Lot No: 3498 T,3) were obtained from Cell Signaling Technology (Danvers, MA, USA). Antibodies against microtubule-associated proteins 1A/1B light chain 3B (LC3B) (ab48394, Lot No: GR3199111-5), cleaved caspase-3 (ab13585, Lot No: GR274345-1), Beclin-1 (ab62557, Lot No: GR3205839-5), LY294002 (PI3K inhibitor) (ab146593, Lot No: APN08061-6–12-S), p62 (ab56416, Lot No: GR3234289-1), and GFAP (ab7260, Lot No: GR3249186-1) were purchased from Abcam (Cambridge, UK). mTOR siRNA (sc-35409, Lot No: G0518) was obtained from Santa Cruz Biotechnology (Dallas, TX, USA). Alexa Fluor 488-labelled anti-rabbit antibody (Abcam, UK, ab150077, Lot No: GR3245102-1) was used for immunofluorescence. Tetrazolium chloride (TTC) and NeuN (MAB377, Lot No: 2919676) were obtained from Sigma (Shanghai, PR China), whereas Evans blue was bought from Urchem (Shanghai, PR China).

### Animals

Male Sprague Dawley rats were obtained from Shandong Luye Pharmaceutical Company (Shandong, PR China, Permit Number: SCXK 2018 0003). The animals were housed with the following conditions: 22 °C ± 2 °C, 50% ± 10% relative humidity, 12 h light/dark cycle, with food and water ad libitum. The Guide for the Care and Use of Laboratory Animals (NIH Publications No. 80–23, USA), revised in 1996, was followed when conducting all animal studies. The studies were also carried out in compliance with the ARRIVE guidelines and the animal welfare compliance Guide for the Care and Use of Laboratory Animals (8thed., 2011). All experiments and procedures were conducted following the Animal Care Guidelines of the Animal Experiment Committee of Binzhou Medical University (China; authorization number BYLY 2015–74).

### Induction of cerebral ischemia by ischemia/reperfusion procedure

After acclimatization for one week, rats within weight of 270–300 g were injected intramuscularly with ketamine 100 mg/kg and xylazine 10 mg/kg to achieve anesthesia. The rectal temperature was kept constant throughout the surgical procedure, and the rectal temperature was recorded and maintained at 37 °C. Middle cerebral artery occlusion (MCAO) was performed as described elsewhere [19]. In brief, occlusion of the left common carotid artery was performed, and then the branches of the external carotid artery were isolated and divided. The internal carotid artery was traced rostrally, and the 4–0 filament (a diameter of 0.25 mm), which had a tip diameter of 0.34 mm, was used to form a globular stopper and inserted into the internal carotid artery, and the filament was then pushed forward until resistance was observed. The filament was removed after 90 min.

One hundred rats were divided into into five groups (Group I ~ V, half animals for Neurological Severity Score (mNSS) and infarct volume detection, the other animals for BBB detection, western blotting and immunohistochemical staining detection) with 20 rats per group: I) non-ischemia/reperfusion (IR) (sham) group; II) I/R group (treated with saline only); III) 2.5 mg/kg cornin group; IV) 5 mg/kg cornin group; and V) cornin 10 mg/kg group. After reperfusion for 15 min, cornin were intravenously injected (*i.v.*) at various dosages via the tail vein. After 24 h of reperfusion, rats neurological deficit scores, as well as the blood–brain barrier (BBB) permeability were performed [19, 20]. The rats were placed in a clear glass box of approximately 10 L with a CO_2_ perfusion rate of 2 L per minute for euthanized. The brains were collected for brain infarct volume determination after transcardial perfusion with phosphate-buffered saline (PBS), the brains corresponding to the ischemic core, peri-infarct cortex rat brain tissues were collected and cutted into two pieces, one piece stored at -80 °C and utilized in western blotting, the other piece was fixed with postfixed with 4% paraformaldehyde in PBS overnight at 4 °C for subsequent immunohistochemical staining in the second group.

For the mechanism study, 50 rats were divided into 5 groups [21]: VI) non-ischemia/reperfusion (IR) (sham) group; VII) I/R group (treated with saline only); VIII) 10 mg/kg cornin group; IX) 10 mg/kg cornin combined with LY294002 group; and X) LY294002 group. After 24 h of reperfusion, the rats were euthanized with carbon dioxide (CO_2_). The brains corresponding to the peri-infarct cortex were collected and utilized for Western blot analysis.

### Assessment of neurological deficit scores

Neurological deficit scores were assessed at 24 h after reperfusion using the flowing method [19]. Briefly, neurological function was graded on a scale of 0 to 18 (normal score, 0; maximal deficit score, 18). The mNSS is a composite of sensory, reflex, and balance tests. In the severity scores of injury, 1 score point is awarded for the inability to perform the test or for the lack of a tested reflex. Thus, a higher score corresponds to a more severe injury.

### Evans blue extravasation and measurement of infarct volume

At 24 h post-reperfusion, rats were sacrificed using deep anesthesia, and the brains were immediately collected. Five coronal sections of the brain with 2.0-mm thickness were prepared and incubated in a 2% 2,3,5-Triphenyl-2H-tetrazolium chloride (TTC) solution at 37 °C for 20 min. Infarct brain tissues appear white, while normal brain tissues appear red. The sections were digitized, and the infarct areas were measured using Image Pro Plus software to calculate the percentage of cerebral infarct areas [22].

At 24 h post reperfusion, 0.1 ml of 4% Evans blue mixed in 0.9% saline was administered intravenously. A quantitative assay using Evans blue was performed to determine blood–brain barrier (BBB) leakage based on a method described previously [23].

### Western blot analysis in vivo

Extraction of total protein from the ischemic penumbra in the cerebral cortex was performed in the experiment. The samples were homogenized with lysis buffer containing a protein inhibitor, the supernatant was obtained, and the amount of protein in the supernatant was determined using the BCA assay. Approximately 50 μg tissue protein samples were separated by SDS-PAGE and then analyzed by western blotting using specific antibodies against p-mTOR, mTOR, Bcl-2, Bax, p-Akt, Akt, Beclin-1, GFAP, p62, LC3B, cleaved caspase-3, and GAPDH at 4˚C overnight. A Gel Doc2000 system (Bio-Rad, USA) was used for scanning and quantification of strip optical density. The data were normalized to GAPDH.

### PI3K inhibitor LY294002 intervention experiment

Rats were injected intramuscularly with ketamine 100 mg/kg (i.m.) and xylazine 10 mg/kg (i.m.) to achieve anesthesia. The CI/R rats were injected with LY294002 via the lateral ventricle approximately 30 min prior to reperfusion. LY294002 was dissolved in dimethyl sulfoxide (DMSO) immediately before the operation to obtain a final concentration of 10 mM, and 10 μL of LY294002 solution was injected into the rat’s lateral ventricle using a brain stereotaxic device (Reward, Shenzhen, China) [21]. The stereotactic intracerebroventricular (ICV) injection site was selected from the following areas of the bregma: anterior and posterior, 0.8 mm; lateral, 1.5 mm; and depth, 3.5 mm.

### Immunohistochemistry staining

The ischemic penumbra in the cerebral cortex was fixed in 4% paraformaldehyde for 48 h, then dehydrated, embedded in paraffin, and sectioned at 4-μm thickness. The histological sections were placed on glass slides coated with polylysine, deparaffinized in xylene, and rehydrated across an alcohol gradient, then stained by immunohistochemistry. The expressions of neuronal nuclei (NeuN), GFAP, LC3-II, and Beclin-1 were detected. Positive expression was assessed using an Image-Pro Plus analysis system.

### Cell culture

Human glioma U87 cells were obtained from the Cell Bank of the Chinese Academy of Sciences. The U87 cells were cultured in dulbecco's modified eagle medium (DMEM) supplemented with 10% fetal bovine serum (FBS) and antibiotics consisting of 100 μg/ml streptomycin and 100 U/ml penicillin at 37 °C and 5% CO_2_. The cells were subcultured upon reaching 80% confluency.

### *In vitro* oxygen glucose deprivation/reperfusion model

To simulate *in vitro* glucose and oxygen deprivation, the U87 cells were subjected to a hypoxic environment for three hours. An *in vitro* oxygen glucose deprivation/reperfusion (OGD/R) model was established as in previous study [22]. Before hypoxia, pretreatment of cells was performed using various cornin concentrations (10, 30, 100, 300 and 1000 nM) for 48 h. A negative control was prepared using a normal culture using DMEM with 1% FBS in 5% CO_2_ air and 20% O_2_, whereas a hypoxia solution culture was employed as the control.

### Cell viability assays

The U87 cells were kept in the hypoxic solution with or without cornin for three hours, and then cell viability was evaluated using an cell counting Kit-8 (CCK-8) assay. Cells at a density of 1 × 10^5^ cells/well were seeded into 96-well plates. The CCK-8 solution (10 μL/well) was placed into each well and further cultured at 37˚C for 4 h. Absorbance at 450 nm was then assessed with a Molecular Devices SpectraMax M5 instrument (Sunnyvale, CA, USA).

### Western blot analysis *in vitro*

The U87 cells were cultured for 48 h, then PI3K inhibitor LY294002 intervention was performed, and mTOR siRNA was added for transfection. Treated cells were followed by washing three times with ice-cold phosphate buffered sline (PBS) and lysing using RIPA lysis buffer (Beyotime, PR China) containing 1 mM PMSF (Sigma-Aldrich). Similar amounts of cell protein (30 μg) were separated by SDS-PAGE, and then analyzed by western blotting using specific antibodies against p-mTOR, mTOR, Beclin-1, p62, LC3B, p-Akt, and Akt, with β-actin as reference, at 4˚C overnight. The strips were scanned, and optical densities were quantified using a Gel Doc2000 (Bio-Rad, USA). Data normalization was performed using β-actin.

### Determination of apoptosis

An Annexin-V FITC apoptosis detection kit was employed to identify apoptotic cells. First, the cells were collected, washed, and then cultured in the dark in the presence of Annexin-V FITC, as well as propidium iodide, at 4 °C for 30 min, followed by analysis using flow cytometry (BD Biosciences, Franklin Lakes, NJ, USA) [22].

### Immunofluorescence detection

The distribution and expression of LC3B were detected by immunofluorescence in U87 cells. After cell treatment, imaging was conducted under a fluorescence microscope (Olympus IX73, Japan).

### Statistical analysis

All experiments were performed in triplicate. The data from the experiments were expressed as the mean ± SD. Statistical analysis was performed using one-way ANOVA followed by Tukey’s test. Differences with *P* < 0.05 were considered statistically significant.

## Results

### Cornin improves functional recovery, decreases infarct volume and BBB leakage after CI/R

There were 5, 3, 3 and 2 and 2 rats (MCAO, cronin 2.5, 5 and 10 mg/kg groups, respectively) found died 24 h after the MCAO. Behavioral assessment is an important index for the recovery of neurological function. The mNSS scores in the MCAO group were higher compared with the sham operation group (*P* < 0.01), whereas the scores were significantly (*P* < 0.01) reduced in cornin-treated rats at doses of 2.5, 5 and 10 mg/kg (Table 1).

**Table 1:** Effect of cornin on survival rate, infaract volume, and neurological scores post CI/R

Group	Survival rate	Infarct volume (%)	Neurological scores
Sham	20/20	0 ± 0	1.8 ± 0.57
MCAO	15/20	30.6 ± 4.5^##^	15.1 ± 2.8^##^
Cornin (2.5 mg/kg)	17/20	28.3 ± 3.0	12.7 ± 3.8
Cornin (5 mg/kg)	17/20	25.6 ± 5.3	11.0 ± 2.8^*^
Cornin (10 mg/kg)	18/20	20.8 ± 4.5^**^	10.4 ± 2.8^**^

Data are presented as the mean ± SD. Comparison of neurological deficit scores among groups using Kruskal Wallis followed by Dunn’s test.

^*^P < 0.05

^**^P < 0.01 *vs*. MCAO group

^##^*P* < 0.01 *vs*. Sham group

**Fig. 1:**
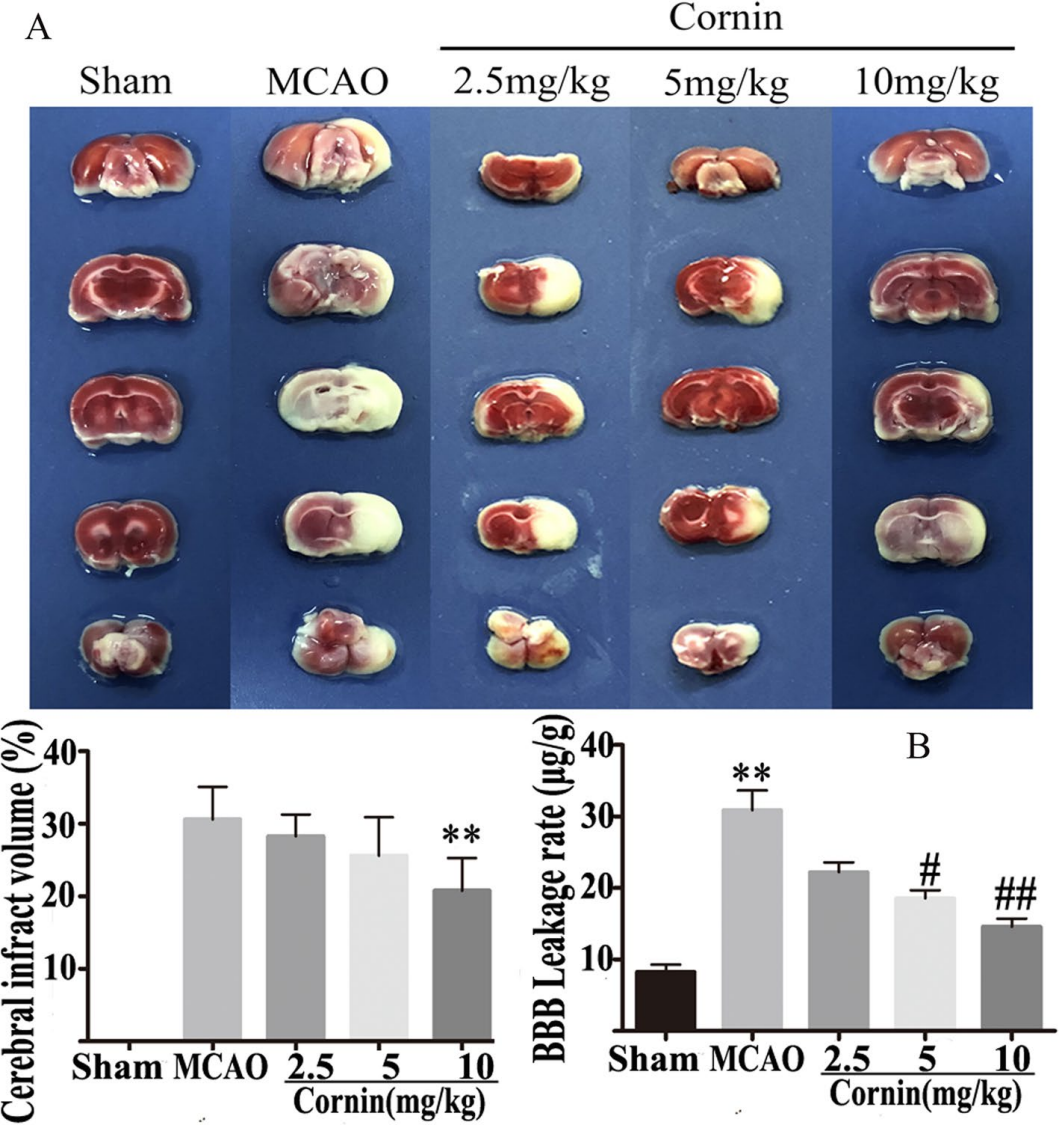
Effects of cornin on infarct volume and leakage in the blood–brain barrier after rats CI/R injury. **A** Representative images of 2,3,4-triphenytetrazolium-chloride (TTC) staining and relevant quantitative analysis. The normal brain area appeared red, while the infarcted area was white. Quantitative analysis of cerebral infarct areas; the data are expressed as the mean ± SD, (*n* = 6). ^**^P < 0.01 *vs.* MCAO group. **B** The permeability of the blood–brain barrier was quantitatively assessed by measuring the extravasated Evans blue. The data are expressed as the mean ± SD, (*n* = 6). ^**^P < 0.01 *vs.* the sham group; ^#^*P* < 0.05, ^##^*P* < 0.01 *vs.* MCAO group

The infarct volume was decreased in cornin-treated groups at doses of 2, 5, and 10 mg/kg in a dose-dependent manner, and was significantly decreased at 10 mg/kg compared to the MCAO group (*P* < 0.01) (Fig. 1A). Ischemia damaged the function of the BBB, and significantly increased the permeability of the BBB in MCAO rats. BBB leakage was significantly decreased in the 10 mg/kg cornin group compared with rats in the MCAO group (*P* < 0.01) (Fig. 1B). These findings indicate that cornin alleviates CI/R injury.

### Cornin inhibits apoptosis and autophagy by activating the PI3K/Akt/mTOR pathway after CI/R in rats

**Fig. 2:**
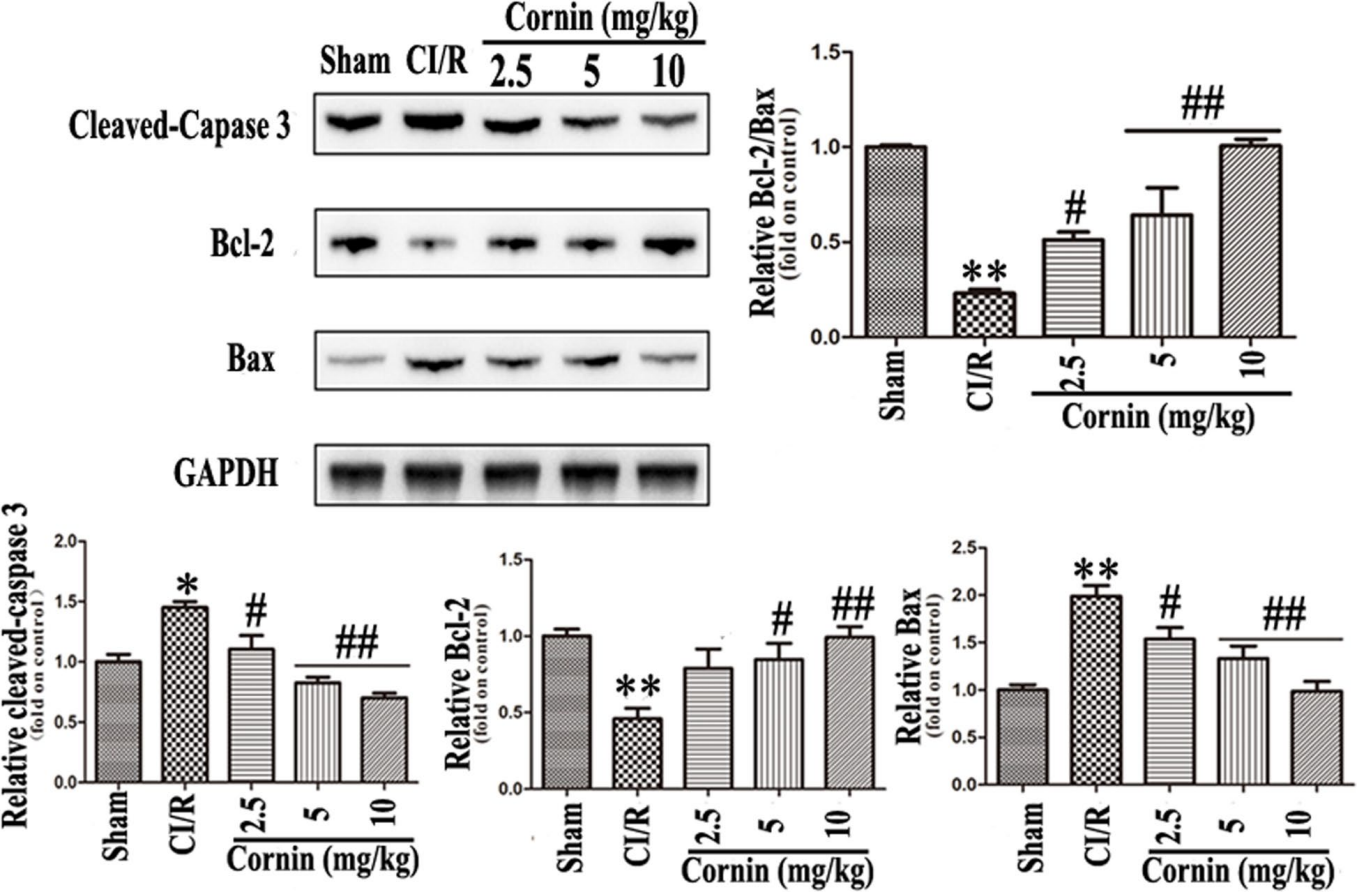
Effect of cornin on apoptosis post rats CI/R injury. Apoptosis-associated proteins were evaluated using western blot analysis, and the full-length blots/gels are presented in Figure S1. After CI/R, rats were treated with cornin (2.5, 5, 10 mg/kg) for 24 h, and equal amounts of tissue lysate protein (50 μg) were separated by SDS-PAGE and analyzed by western blotting using specific antibodies. The expression levels of cleaved caspase-3, Bcl-2, and Bax were determined and presented as the mean ± SD, (n = 3). ^*^P < 0.05, ^**^P < 0.01 *vs.* the sham group; ^#^*P* < 0.05, ^##^*P* < 0.01 *vs.* CI/R group

**Fig. 3:**
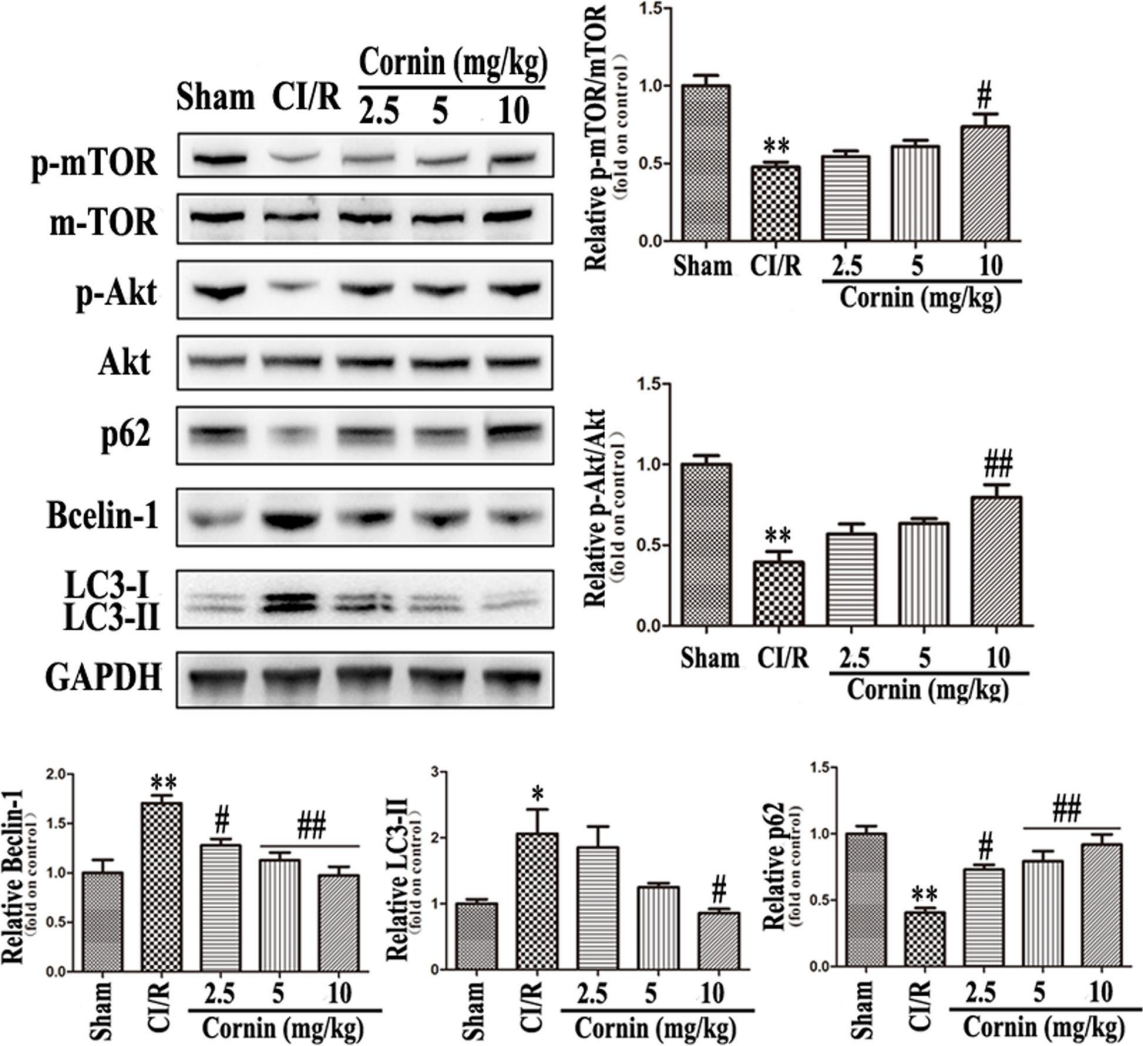
Effect of cornin on autophagy post rats CI/R injury through the PI3K/Akt/mTOR signaling pathway. Autophagy-associated proteins were assessed by western blotting using western blot analysis, and the full-length blots/gels are presented in Figure S2. After CI/R, rats were treated with cornin (2.5, 5, 10 mg/kg) for 24 h, and equal amounts of tissue lysate protein (50 μg) were separated by SDS-PAGE and analyzed by western blotting using specific antibodies. LC3-II, p62, Beclin-1, p-mTOR, and p-Akt expression levels were quantified and are represented as the mean ± SD, (*n* = 3). ^*^P < 0.05, ^**^P < 0.01 *vs.* the sham group; ^#^*P* < 0.05, ^##^*P* < 0.01 *vs.* CI/R group

To detect the effects of cornin on autophagy and apoptosis after CI/R injury in rats, Bcl-2, Bax, cleaved caspase-3, LC3-II (isolation from LC-B protein bands), Beclin-1, and p62 protein levels were assessed. Western blotting revealed that Bax, cleaved caspase-3, LC3-II, and Beclin-1 expression levels were significantly upregulated (*P* < 0.05), and Bcl-2 and p62 were significantly decreased (*P* < 0.01) in CI/R group compared with sham group. However, Bax, cleaved caspase-3, LC3-II, and Beclin-1 levels were significantly downregulated (*P* < 0.01), while Bcl-2 and p62 were significantly upregulated in CI/R rats treated with cornin at 2.5, 5, or 10 mg/kg (*P* < 0.01) (Fig. 2 and Fig. 3). In contrast, the expression of Bax and Bcl-2 was reversed by the PI3K inhibitor LY294002 (Fig. 5B). The expression of p-mTOR and p-Akt was also markedly reduced (*P* < 0.01, compared with sham group) after CI/R, and significantly increased (*P* < 0.01, compared with CI/R group) with 10 mg/kg cornin (Fig. 3).

**Fig. 4:**
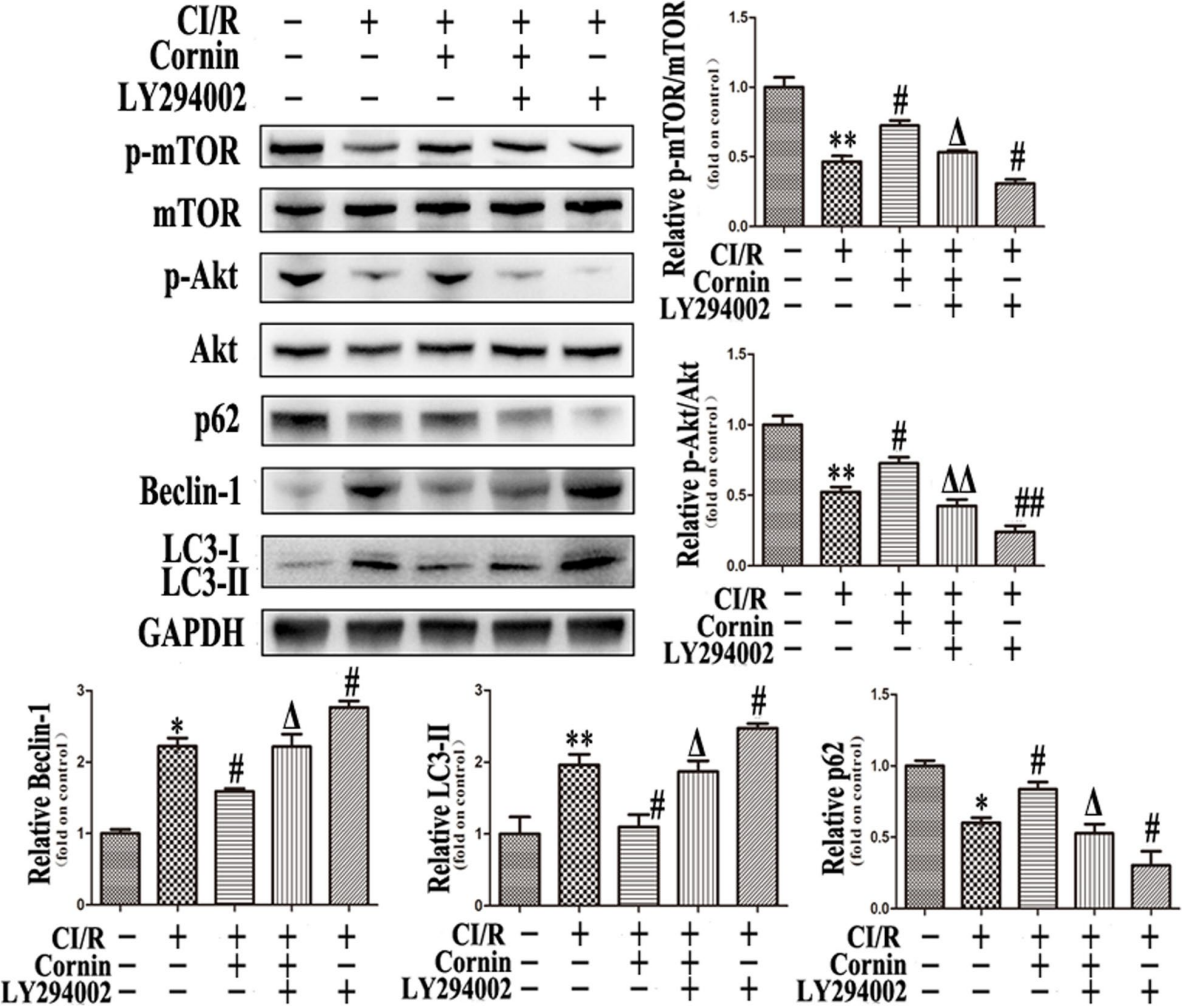
Effect of PI3K inhibitor LY294002 on autophagy post rats CI/R injury. The rats were injected with PI3K inhibitor LY294002 (10 mM) before cornin (10 mg/kg) treatment. Autophagy-associated proteins were assessed by western blotting using western blot analysis, and the full-length blots/gels are presented in Figure S3. LC3-II, p62, Beclin-1, p-mTOR, and p-Akt expression levels were quantified and are represented as the mean ± SD, (*n* = 3). ^*^P < 0.05, ^**^P < 0.01 *vs.* the sham group; ^#^*P* < 0.05, ^##^*P* < 0.01 *vs*. CI/R group; ^Δ^*P* < 0.05, ^ΔΔ^*P* < 0.01 *vs.* cornin 10 mg/kg group

PI3K inhibitor LY294002 was used to further verify whether cornin affects autophagy induced by CI/R injury through the PI3K/Akt/mTOR signaling pathway. Western blotting revealed that the expressions of p-mTOR, p-Akt, and p62 were all partially blocked by LY294002, in contrast, LC3-II and Beclin-1 were increased abundantly (Fig. 4). These results show that inhibiting the expression of PI3K also inhibits mTOR and activated excessive autophagy of the cells. Therefore, cornin may reduce the autophagy induced by CI/R injury through PI3K/Akt/mTOR pathway.

### Protective effects of cornin on CI/R in immunohistochemistry-related protein

**Fig. 5:**
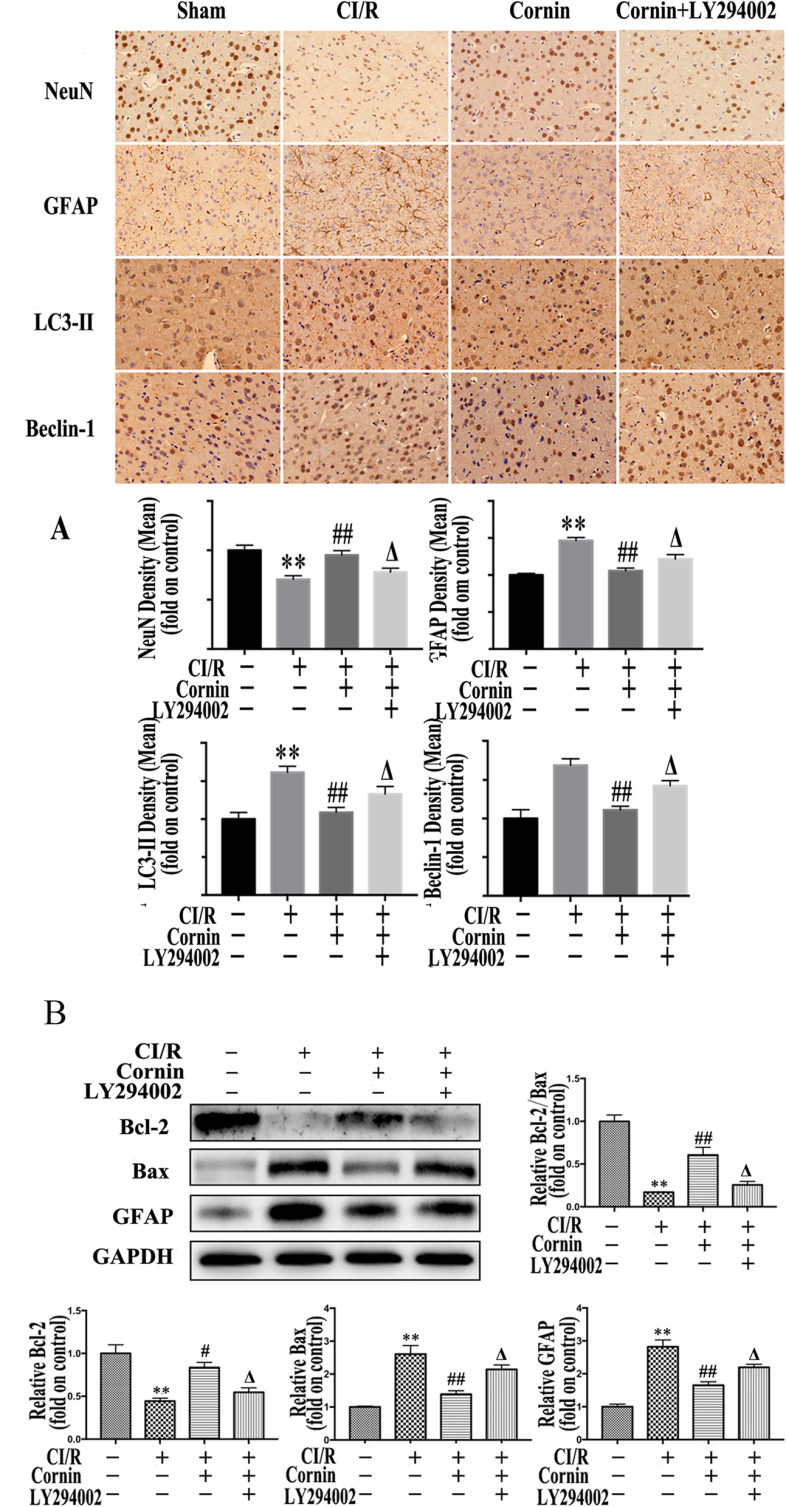
Protective effects of cornin on rats CI/R inury assessed by immunohistochemistry and western blot. **A** After 24 h of CI/R injury, the number of cells positive for NeuN, GFAP, LC3-II, and Beclin-1 in the sham group, CI/R group, cornin (10 mg/kg) group, and cornin + LY294002 group was quantitatively evaluated by immunohistochemistry. The magnification was × 200. The data are presented as the mean ± SD, (*n* = 3). ^**^P < 0.01 *vs.* the sham group; ^##^*P* < 0.01 *vs.* CI/R group; ^Δ^*P* < 0.05 *vs.* cornin 10 mg/kg group. **B** GFAP, Bax and Bcl-2 was assessed by western blotting using western blot analysis, and the full-length blots/gels are presented in Figure S4, and the expression levels were quantified and are represented as the mean ± SD, (*n* = 3). ^**^P < 0.01 *vs.* the sham group; ^##^*P* < 0.01 *vs.* CI/R group; ^Δ^*P* < 0.05 *vs.* cornin 10 mg/kg group

The expressions of NeuN, GFAP, LC3-II, and Beclin-1 at 24 h after CI/R injury were measured by immunohistochemistry. The expression of GFAP was lower in the sham operation group, and brown astrocytes were readily observed in the model group. Compared with the CI/R group, the number of positive astrocytes significantly decreased in cornin treated group (*P* < 0.01). The PI3K inhibitor LY294002, combined with cornin, significantly increased the number of positive astrocytes (*P* < 0.05) (Fig. 5A). At the same time, the expression of GFAP protein was detected by western blotting. The GFAP expression was significantly reduced after cornin treatment (compared with CI/R group, *P* < 0.01). Also, the PI3K inhibitor LY294002, when combined with cornin, significantly increased the expression of GFAP protein (*P* < 0.05) (Fig. 5B).

In the CI/R group, most of the neurons showed nuclear pyknosis, which is an indicator of injury induced by CI/R that was rarely observed in the cornin group, due to the neuroprotective effect of cornin. However, PI3K inhibitor LY294002 could decrease the protective effect of cornin (*P* < 0.05). The number of positive cells for Beclin-1 and LC3-II in the CI/R group was higher than that in the sham group, and the number of positive cells in the cornin group was significantly decreased compared to the CI/R group (*P* < 0.01). The combination of LY294002 and cornin increased the number of positive cells (*P* < 0.05) (Fig. 5A).

### Cornin improves cell viability on oxygen glucose deprivation/reperfusion induced death in U87 cells

**Fig. 6:**
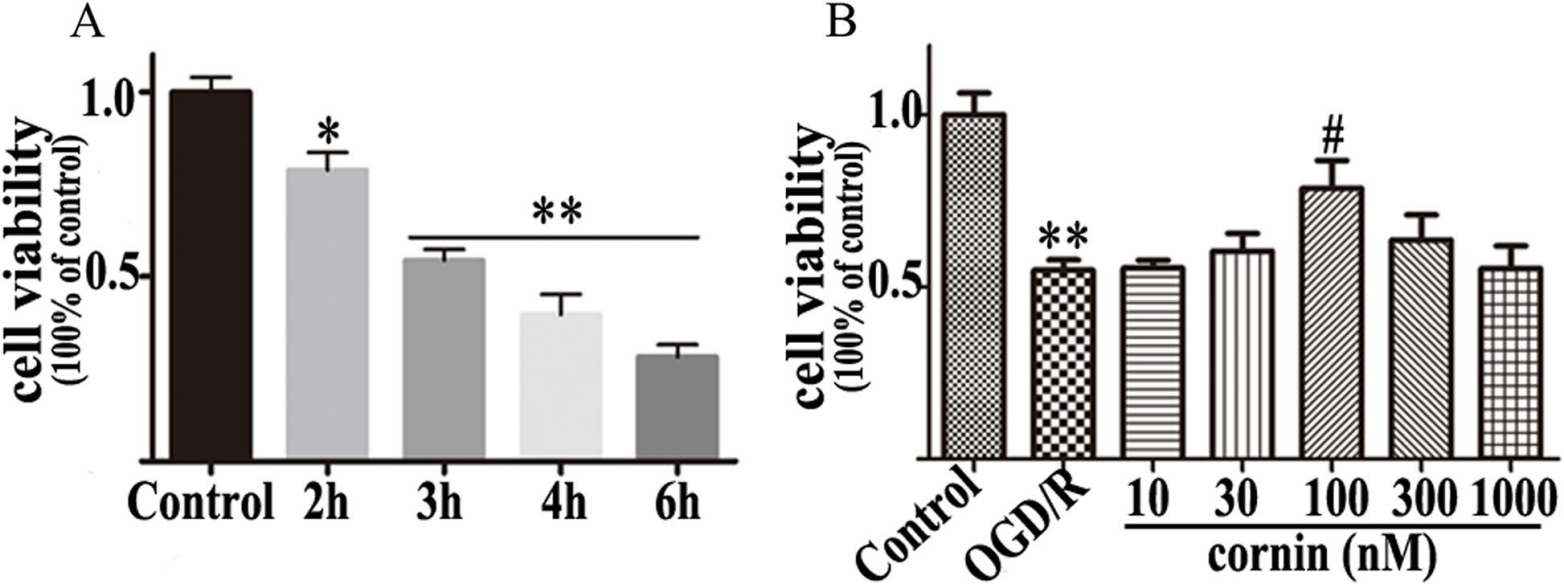
Protective effect of cornin in OGD/R-induced U87 cell death. **A** The viability of U87 cells exposed to OGD/R at different durations as assessed using the CCK-8 assay. **B** U87 cells were pre-incubated with different concentrations of cornin for 48 h, then underwent oxygen–glucose deprivation for 3 h, followed by reoxygenation for 24 h to detect cell viability changes. Data are expressed as the mean ± SD, (*n* = 6). Significance was assessed by a one-way ANOVA followed by Dunnett’s test. ^*^P < 0.05, ^**^P < 0.01 *vs*. the control group; ^#^*P* < 0.05 *vs.* the OGD/R group

To evaluate the influence of cornin on proliferation of U87 cells after OGD/R injury, the effect of 48-h cornin (10, 30, 100, 300 and 1000 nM) treatment on the viability and morphological changes in OGD/R-damaged was assessed. Compared with normal cultured cells, the viability of the U87 cells after OGD/R was significantly inhibited (*P* < 0.01) (Fig. 6). However, following treatment with cornin (10, 30, and 100 nM), the cell viability was increased in a concentration-dependent manner, and decreased at doses above 100 nM (Fig. 6).

### Cornin inhibits OGD/R-induced U87 cell apoptosis and autophagy

**Fig. 7:**
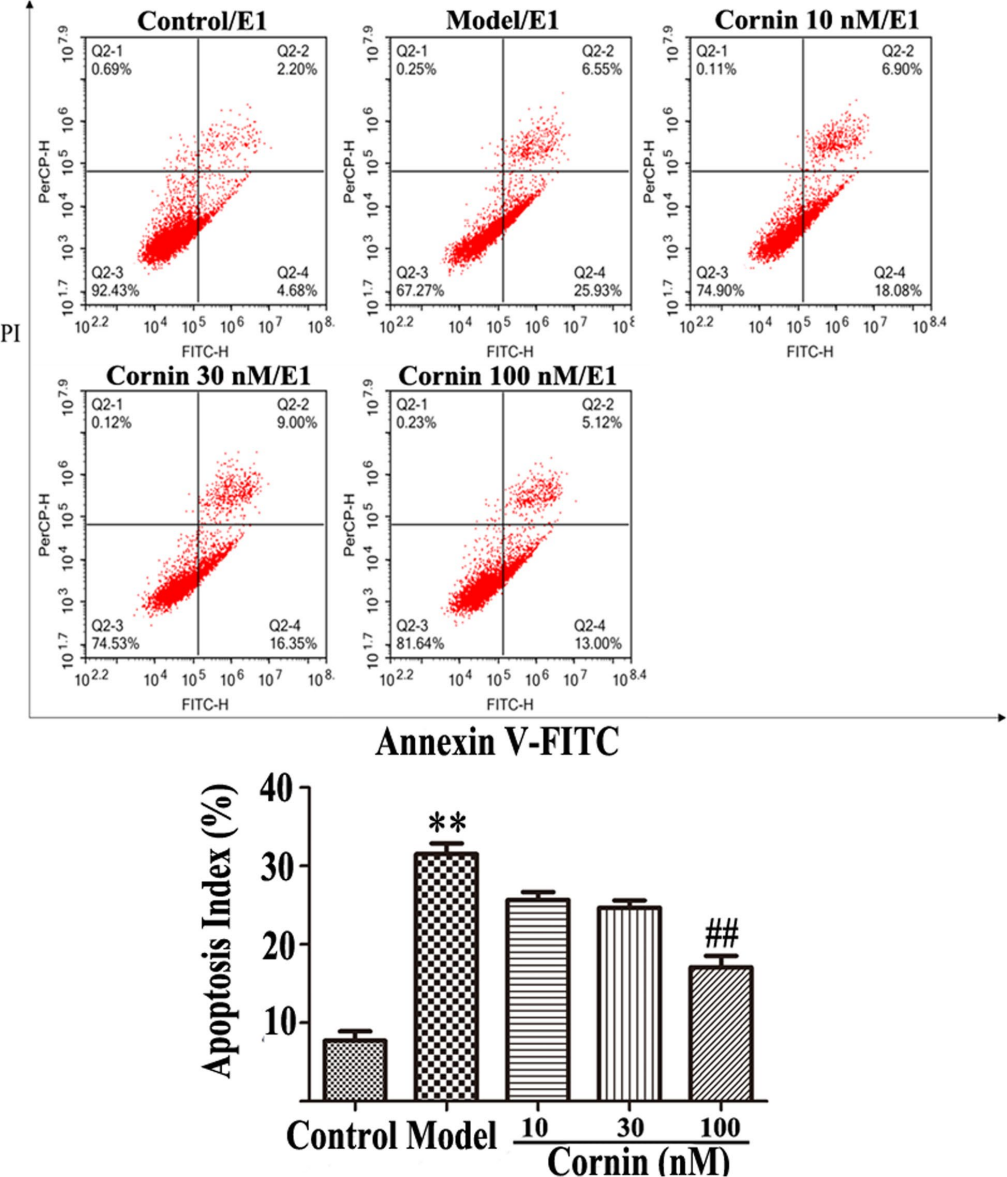
Effect of cornin on U87 cell apoptosis. U87 cells were pretreated with cornin (10, 30, 100 nM) for 48 h, followed by oxygen–glucose deprivation exposure for three hours, and reoxygenation for 24 h. Apoptosis was assess by flow cytometry using Annexin V-FITC/PI fluorescence staining. The sum of the second and fourth quadrants in the figure is the percentage of apoptotic cells. The data of apoptotic cells are expressed as the mean ± SD, (*n* = 3). ^**^P < 0.01 *vs*. the control group; ^##^*P* < 0.01 *vs*. the OGD/R group

Apoptosis in U87 cells was assessed by flow cytometry using AnnexinV-FITC/PI double staining, which included early apoptosis (Annexin V-FITC-positive, PI-negative) as well as late apoptosis (Annexin V-FITC-positive, PI-positive). The proportion of apoptotic cells in the model group was markedly higher than that observed in the normal control group, whereas the proportion of apoptotic cells in the cornin treatment group was reduced in a concentration-dependent manner (Fig. 7).

**Fig. 8:**
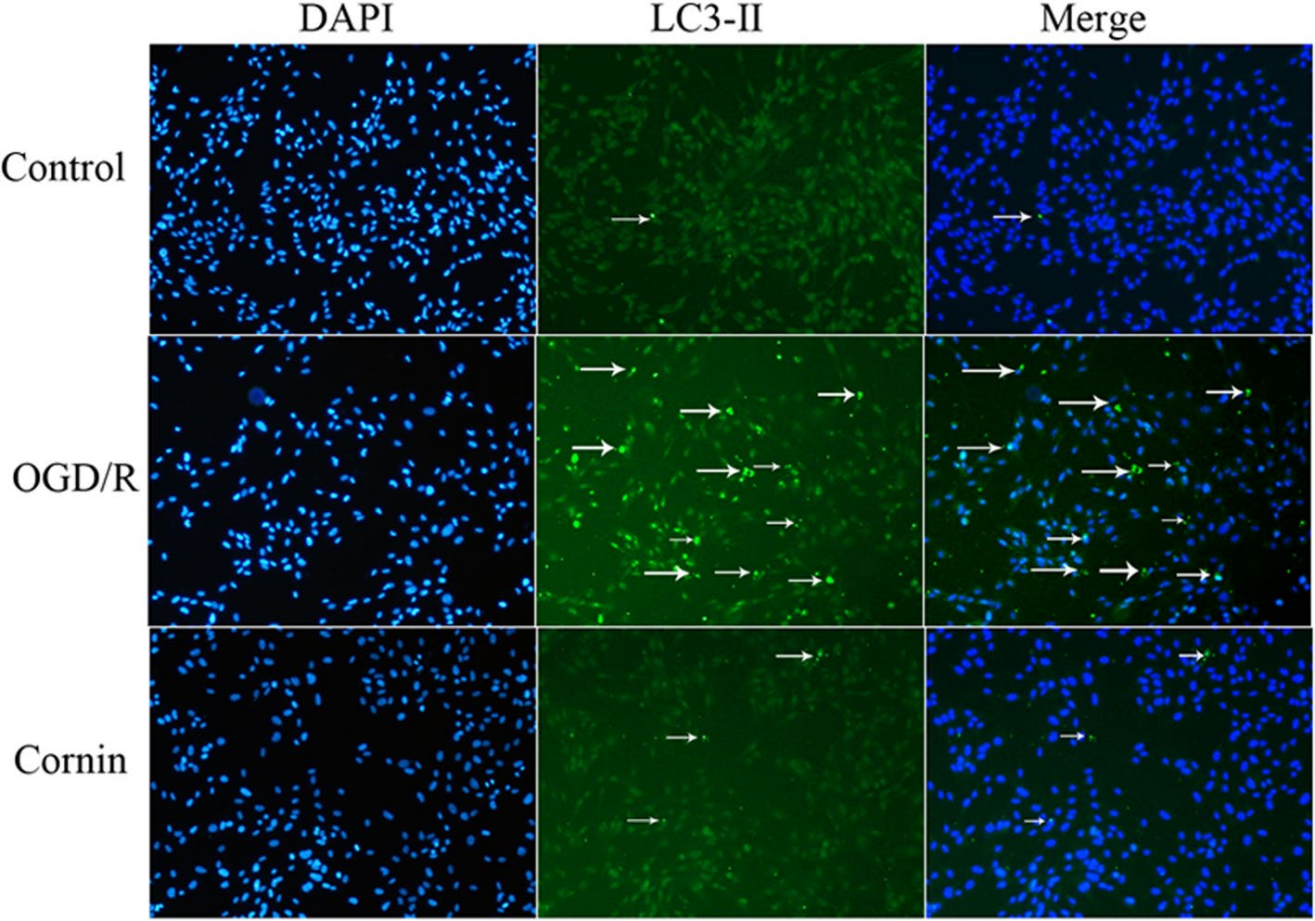
Effect of cornin on U87 cell autophagy. U87 cells were pretreated with cornin (100 nM) for 48 h followed by oxygen–glucose deprivation exposure for three hours, and reoxygenation for 24 h. Immunofluorescence images of U87 cells using the autophagy marker LC3B. The left column shows a nucleus stained with DAPI, the middle column presents immunofluorescence staining using specific markers, and the right column depicts a merged image of the left and middle frames

To confirm whether cornin have protective effect on OGD/R-treated U87 cells, the autophagy-associated protein LC3-II was evaluated. The results revealed that OGD/R significantly upregulated the level of LC3-II compared with normal control group. While cornin significantly inhibited the autophagy activation by decreasing LC3-II expression (Fig. 8).

### Cornin inhibits OGD/R-induced autophagy by activating the PI3K/Akt/mTOR pathway

**Fig. 9:**
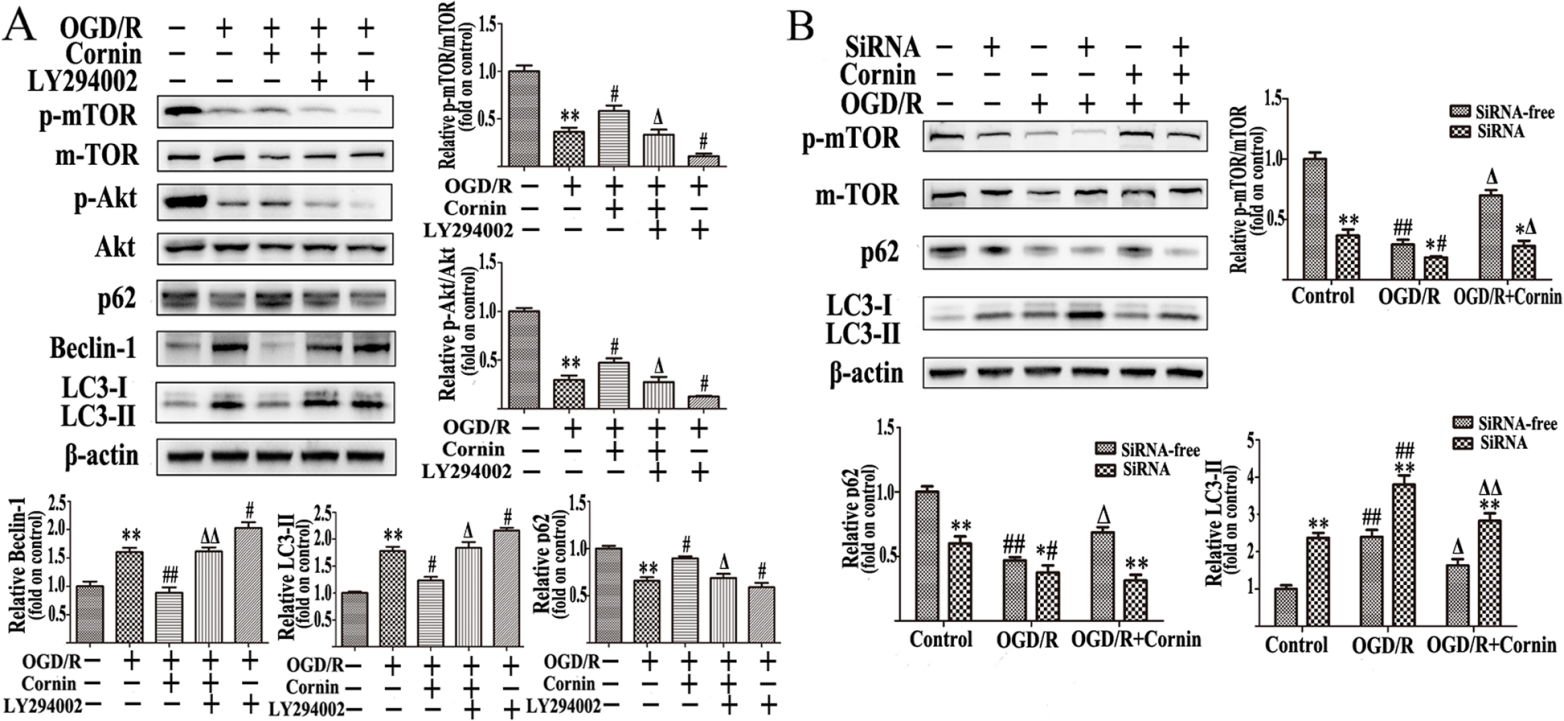
Effect of cornin on autophagy in OGD/R-induced U87 cells through the PI3K/Akt/mTOR signaling pathway. **A** LY294002 (10 μM), a PI3K inhibitor, was used as pretreatment for 6 h, and then incubated with cornin (100 nM) for 48 h. Western blotting of the associated proteins and density analysis were performed, and the full-length blots/gels are presented in Figure S5. The results are presented as the mean ± SD, (*n* = 3). ^**^P < 0.01 vs. the control group; ^#^*P* < 0.05, ^##^*P* < 0.01 *vs.* the OGD/R group; ^Δ^*P* < 0.05, ^ΔΔ^*P* < 0.01 *vs.* 100 nM cornin group. **B** U87 cells were pretreated using mTOR siRNA for 6 h followed by western blotting using western blot analysis, and the full-length blots/gels are presented in Figure S6. The results are presented as the mean ± SD, (*n* = 3). ^*^P < 0.05, ^**^P < 0.01 *vs.* siRNA-free treatment group; and ^#^*P* < 0.05, ^##^*P* < 0.01 *vs.* the control group, respectively; ^Δ^*P* < 0.05, ^ΔΔ^*P* < 0.01 *vs.* OGD/R group

To further verify whether cornin exhibit inhibiting effect on autophagy through the PI3K/Akt/mTOR signaling pathway, LY294002 (a PI3K inhibitor) was used in the 100 nM cornin and OGD/R-treated groups. The protein expression of p-mTOR, p-Akt, and p62 significantly decreased after treatment with LY294002, whereas the protein expression levels of LC3-II and Beclin-1 significantly increased (*P* < 0.05, compared with 100 nM cornin group) (Fig. 9). The application of mTOR siRNA induced a similar effect on the PI3K inhibitor LY294002. After mTOR siRNA transfection, the expression of mTOR and p62 significantly decreased, whereas LC3-II expression significantly increased (Fig. 9). In summary, the results suggest that the astrocyte autophagy inhibitory effect of cornin involves the PI3K/Akt/mTOR pathway.

## Discussion

In a previous study, we have confirmed that cornin exerts a protective effect in CI/R injury, and its mechanism involves antioxidation and promoting angiogenesis effects [18]. In the present study, we confirmed that cornin significantly reduced the cerebral infarct volume (about 9.8% reduction in 10 mg/kg cornin treatment group compared to MCAO group), BBB leakage (48.2%-58.1% inhibition in 5–10 mg/kg cornin treatment group compared to MCAO group) and improved functional recovery (the mNSS scores decreased from 15.1 in MCAO group to 1.04 -11.0 in 5–10 mg/kg cornin treatment group) after CI/R injury in SD rats. The underlying mechanism of action appear to involve prevention astrocytes autophagy induced by CI/R that rely on PI3K/Akt/mTOR signaling pathway.

The BBB is composed of vascular endothelial cells, basement membrane, podocytes and pericytes of astrocytes, microglia, as well as extracellular matrix. Among them, the tight connection between astrocytes and endothelial cells is the basis for the formation of BBB. Astrocytes also play an important role in neuroregulation and synaptic transmission [13]. Autophagy of astrocytes can be robustly activated by oxygen and glucose deprivation or pharmacological inhibition of metabolic stress induced by mTOR [24]. Furthermore, autophagy process is strongly associated with CI/R injury [25]. Studies have confirmed that inhibition of autophagic activity could attenuates CI/R injury [26]. Our results shows that autophagy activation with the increased expression of LC3-II and Beclin-1, as well as decreased expression of P62 in both CI/R rats and OGD/R U87 cells (Fig. 3 and Fig. 9). Cornin plays protective effects in both CI/R-induced autophagy in rats and OGD/R-induced autophagy in U87 cells. *In vitro*, excessive activation of autophagy by OGD/R decreases cell viability, whereas cornin at 10 nM – 100 nM inhibits the over activation of autophagy in a concentration-dependence manner (Fig. 6). The increased LC3-II and Beclin-1 level in U87 cells and brain tissues were reduced and the level of p62 increased with cornin treatment (Fig. 3 and Fig. 9). In addition, the protective effects of cornin was reversed by the autophagy activator LY294002 [27], with LC3-II, Beclin-1 expression significantly increased, and p62 expression significantly decreased (Fig. 4 and Fig. 9). Similarly, the application of mTOR siRNA induced a similar effect on the PI3K inhibitor LY294002 (Fig. 9). *In vivo*, 10 mg/kg cornin significantly decreased infarct volume, BBB leakage after CI/R injury in rats. The mNSS scores behavioral assessment is also indicated cornin could markedly improve the recovery of neurological function. These results indicate that cornin exerts protective effects in CI/R injury, and its mechanism may via inhibiting autophagy activation.

Autophagosomes develop to enclose damaged organelles as well as other macromolecular structures [28]. However, the excessive formation of autophagosomes may aggravate cell injury and lead to autophagic cell death or apoptosis. In the case of CI/R, inflammatory factors secreted by brain tissues activate caspase-3, thereby leading to apoptosis [29]. Pro-apoptotic genes belonging to the Bcl-2 family, including Bax, can similarly activate caspase-3 and induce apoptosis [30]. Autophagy have tightly linked with apoptosis, and the anti-apoptotic protein Bcl-2 is also involved in the autophagy pathway [31]. In addition, Bcl-2 and Beclin-1 interact to target the Beclin-1-dependent autophagy pathway [32]. Our results revealed that the upregulation of cleaved caspase-3 and Bax in CI/R rats brain tissues was effectively reversed by 2.5 – 10 mg/kg cornin treatment, thereby leading to a marked increase in the Bcl-2 to Bax ratio (Fig. 2). These effects were reversed by PI3K inhibitor LY294002 in CI/R rats treated with 10 mg/kg cornin (Fig. 5B). Therefore, cornin may exert its protective effects also by inhibiting the apoptosis induced by CI/R, and its potential mechanism on apoptosis could be associated with a reduction in autophagy as induced by CI/R.

During the autophagy process, the cytoplasmic form of LC3-I is transformed into phosphatidylethanolamine forms to LC3-II, which in turn promotes the formation of autophagosomes. Therefore, the autophagy activation was accompanied by increase of LC3 expression [33]. Beclin-1 is an autophagy-associated protein as well as an important indicator of autophagy [34, 35]. To elucidate the mechanism by which cornin regulates autophagy, the effect of cornin on PI3K/Akt/mTOR pathway activation was evaluated. mTOR is a major regulator of cell growth, proliferation, and autophagy, as well as an important downstream target of the PI3K/Akt signaling pathway [36]. In ischemia and hypoxia, the level of ATP in the brain is reduced because lack of oxygen and nutrition, which in turn inhibiting the expression of mTOR and activating autophagy. Previous studies showed that PI3K/Akt/mTOR signaling is essential to the survival of neurovascular units cerebral ischemia [37]. Our study revealed that the infarct volume as well as the leakage rate of the BBB, was significantly decreased, and the neurological function was significantly improved in CI/R rats after cornin treatment (Table.1 and Fig. 1). These findings confirmed that cornin can alleviates CI/R injury in a dose dependent manner. Simultaneously, cornin treatment markedly increased p-Akt and p-mTOR expression in U87 cells and CI/R rats, and the PI3K inhibitor LY294002 significantly reduced the over expression of p-Akt and p-mTOR. The increased LC3-II and Beclin-1 expression levels in U87 cells and CI/R rats were reduced, and the expression level of p62 increased with cornin treatment. However, these effects were reversed by PI3K inhibitor LY294002 (Fig. 4 and Fig. 9). Similarly, the application of mTOR siRNA induced a similar effect on the PI3K inhibitor LY294002 (Fig. 9). These results indicated that cornin contributes to the regulation of autophagy caused by CI/R, and its potential mechanism may through the PI3K/Akt/mTOR signaling pathway.

In summary, we have demonstrated that cornin plays neuroprotective effect after cerebral ischemia injury in SD rats by preventing astrocytes autophagy induced by CI/R via the PI3K/Akt/mTOR signaling pathway. The astrocytes were protected by autophagy inhibition of cornin, played an important effect on maintaining the integrity of BBB and also regulating neuroregulation and synaptic transmission of nerve cells [13]. Our study lays the foundation for future studies to translate preclinical results to therapeutic efficacy in patients with ischemia stroke. A cascade of inflammatory injuries usually occur after cerebral ischemia injury. Whether cornin have anti-inflammatory effect and its mechanism in CI/R will be carried out in future.

## Supplementary Information

**Additional file 1.** Supplementary materials.

## Data Availability

The datasets generated during and/or analyzed during the current study are available from the corresponding author on reasonable request.
