# Supplementary material for: Cornin protects against cerebral ischemia/reperfusion injury by preventing autophagy via the PI3K/Akt/mTOR pathway
Source: BMC Pharmacol Toxicol. 2022 Oct 24;23:82. doi: 10.1186/s40360-022-00620-3 (PMC9594897; doi:10.1186/s40360-022-00620-3)

Supplementary Information

Title: Cornin protects against cerebral ischemia/reperfusion injury by preventing autophagy via the PI3K/Akt/mTOR pathway

Tianchi Lan[1#], Yangyang Xu[3#], Shucui Li[1], Ning Li [2], Shuping Zhang[1*], Haibo Zhu[2*]

[#] These authors contribute equally to this paper.

[*] Corresponding Author

Shuping Zhang. Email: zsp861122@126.com, Department of Pharmacy, Binzhou Medical University Hospital, Binzhou, Shandong, P.R. China

Haibo Zhu. Email: ythbzhu@163.com, School of Public Health and Management, Binzhou Medical University, Yantai, Shandong, P.R. China

Author details

[1] Department of Pharmacology, Binzhou Medical University, Yantai, Shandong 264003, P.R. China; [2] School of Public Health and Management, Binzhou Medical University, Yantai, Shandong 264003, P.R. China; [3] Department of Pharmacy, Binzhou Medical University Hospital, Binzhou, Shandong 256603.

Fig.s1 Cutting original blots/gels for protein expression detection of cleaved caspase-3, Bcl-2, and Bax after cornin (2.5, 5, 10 mg/kg) treatment for 24 h in CI/R rats. Note: All the SDS-PAGE blots gel cut before the PVDF membrane transfer according to the "Protein Marker", so no full-length blots were provided in this experiment. The gel in the left is high exposure, and the gel in the right is the low exposure for targets protein.

A. Bcl-2

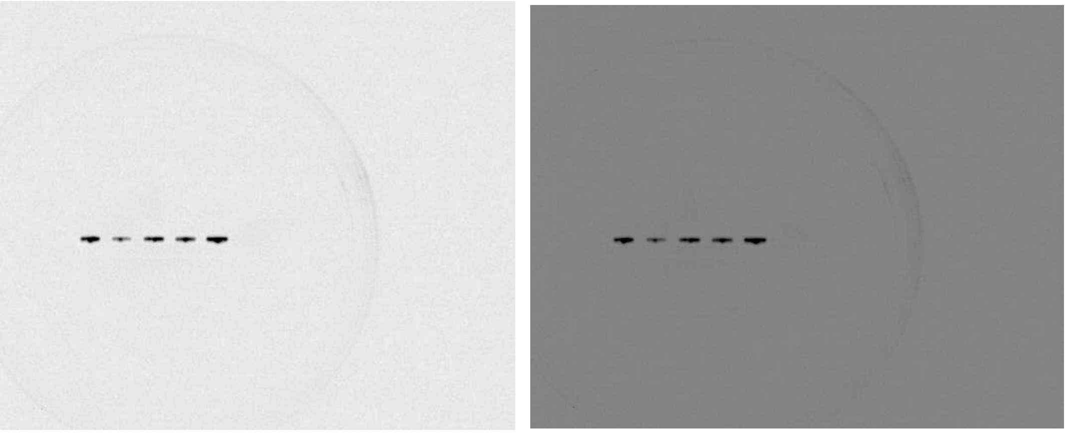

B. Bax

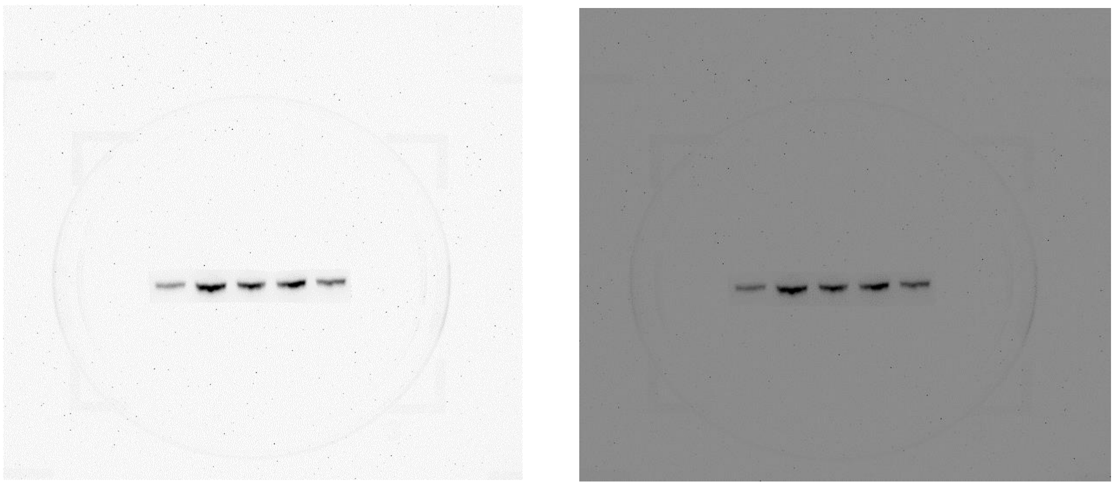

C. Caspase-3

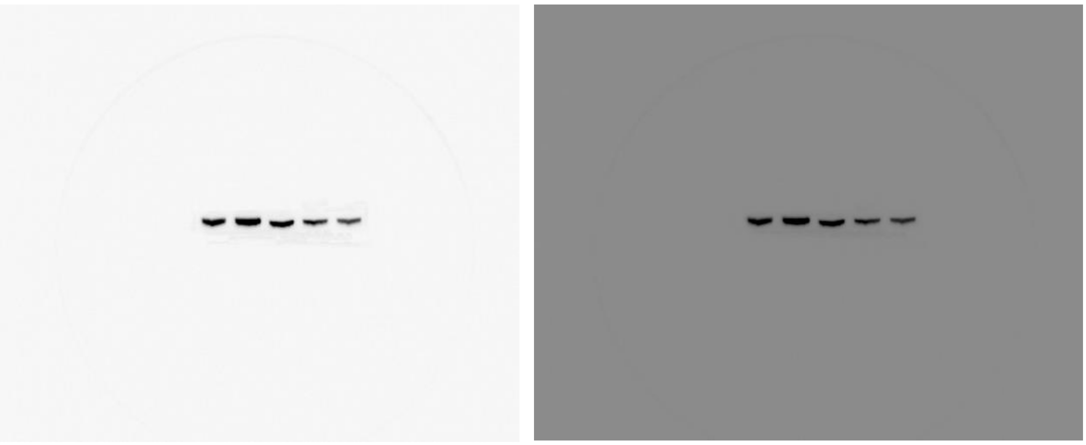

D: GAPDH

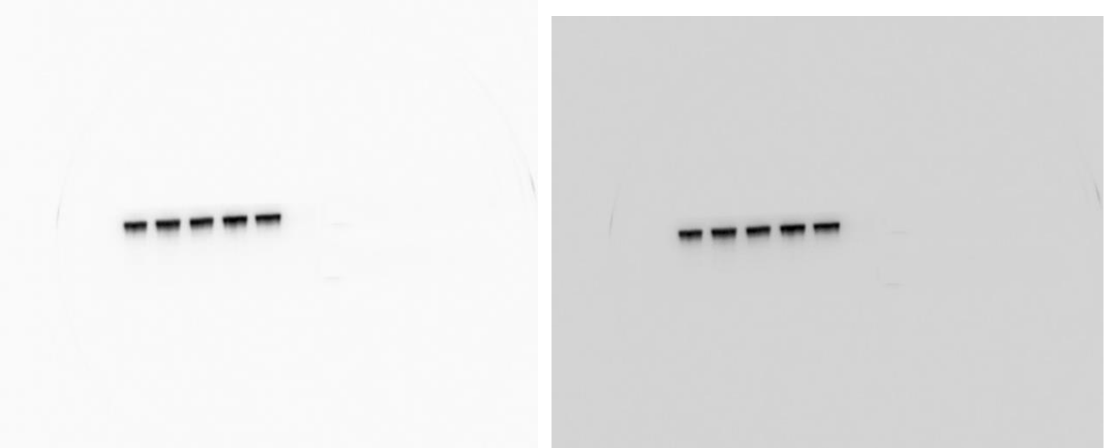

Fig.s2 Cutting original blots/gels for protein expression detection of LC3-II, p62, Beclin-1, p-mTOR, and p-Akt after cornin (2.5, 5, 10 mg/kg) treatment for 24 h in CI/R rats. Note: All the SDS-PAGE blots gel cut before the PVDF membrane transfer according to the "Protein Marker", so no full-length blots were provided in this experiment. The gel in the left is high exposure, and the gel in the right is the low exposure for targets protein.

A: p-mTOR

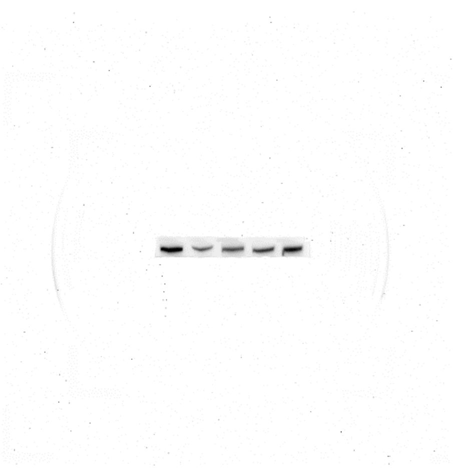

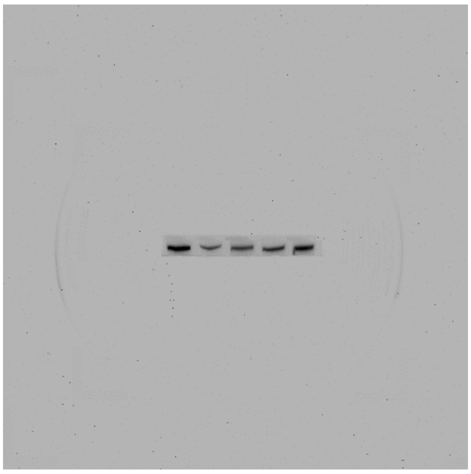

B: mTOR

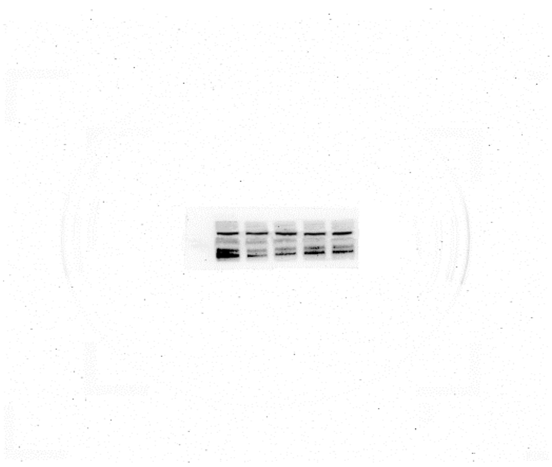

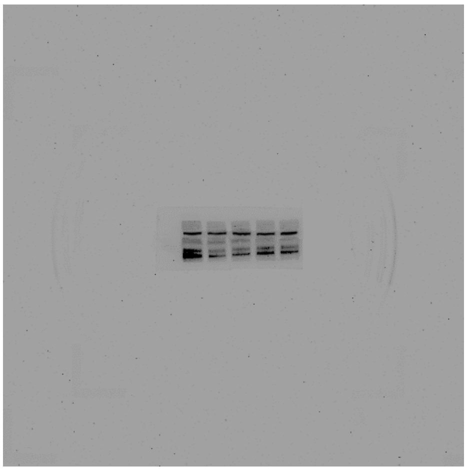

C: p-Akt

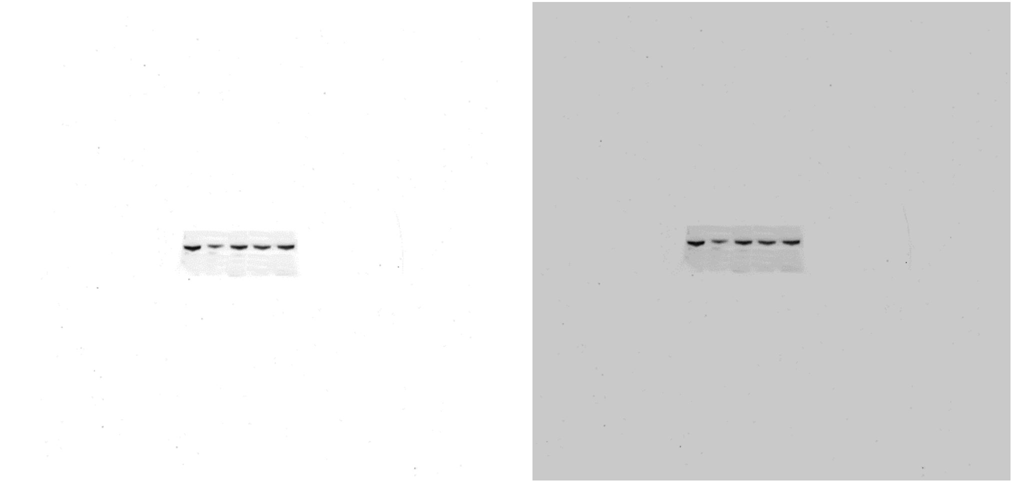

D: Akt

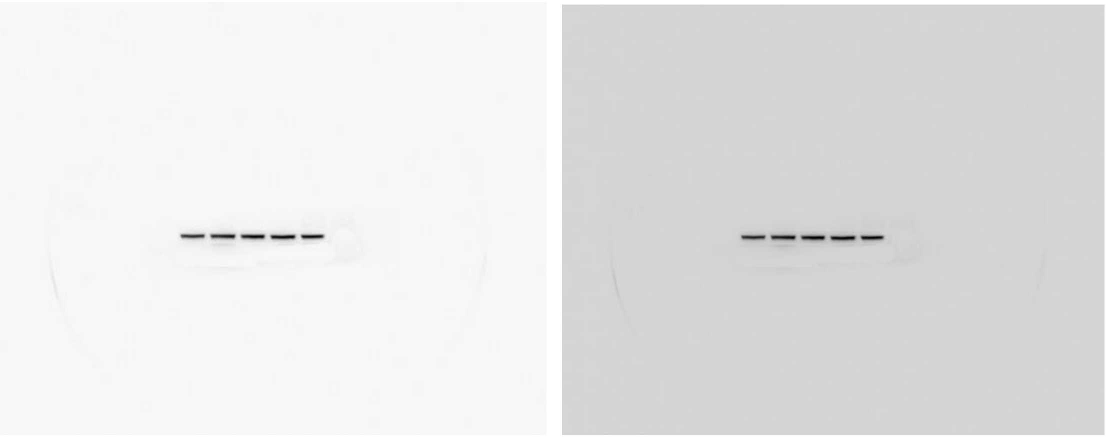

E: p62

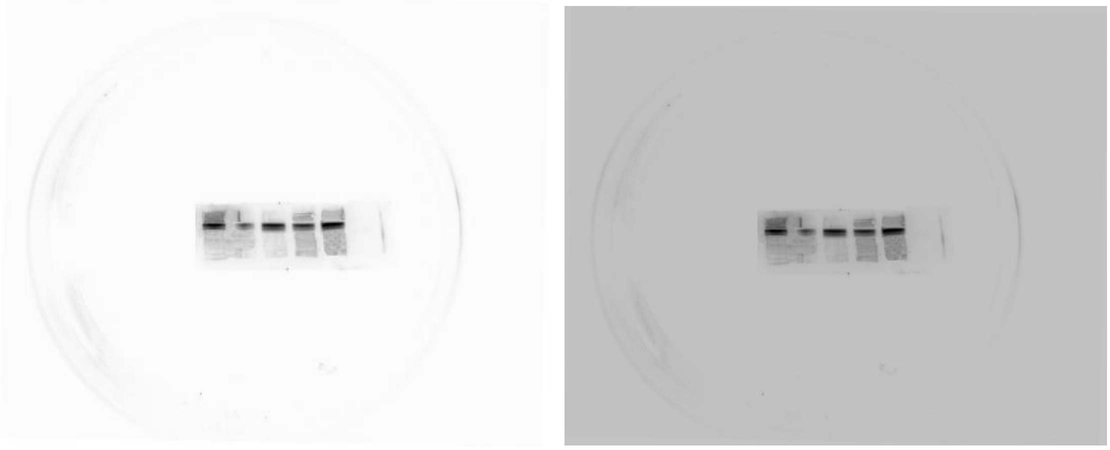

F: Beclin-1

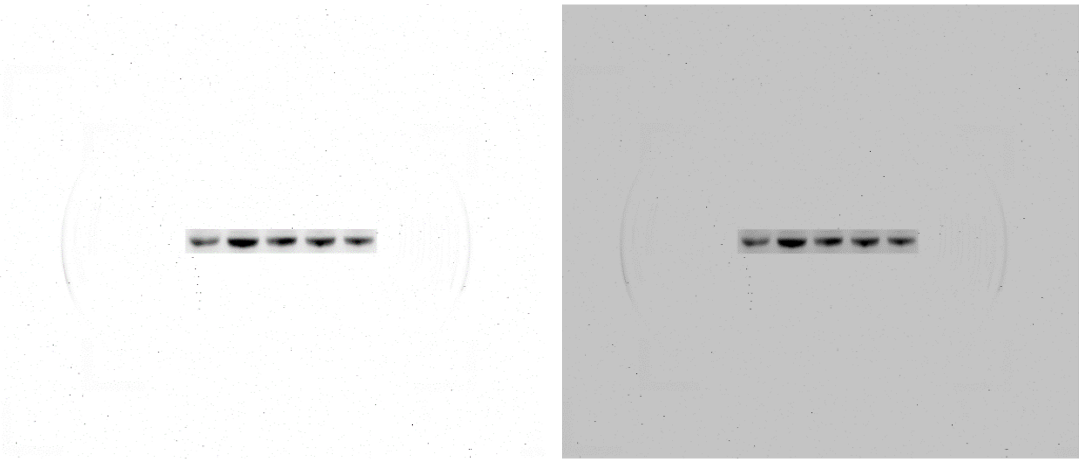

G: LC3-II

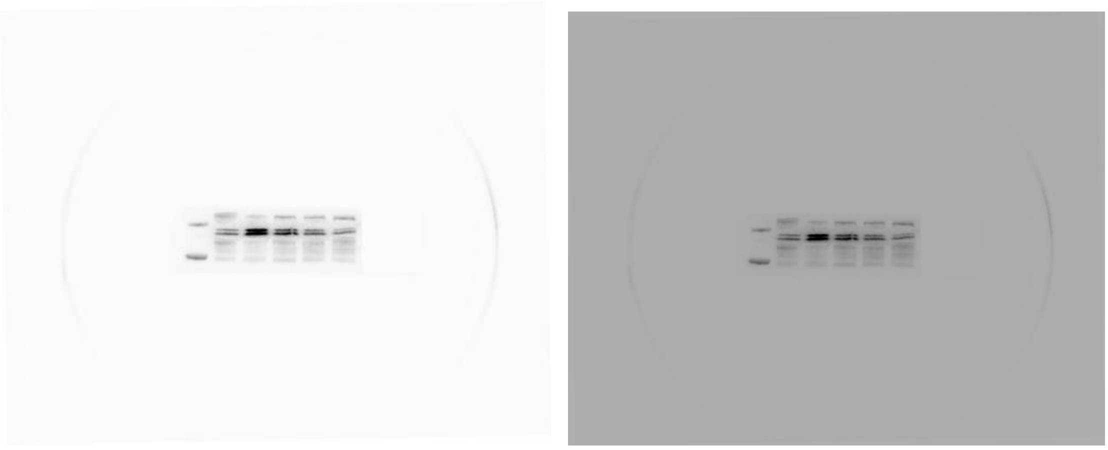

H: GAPDH

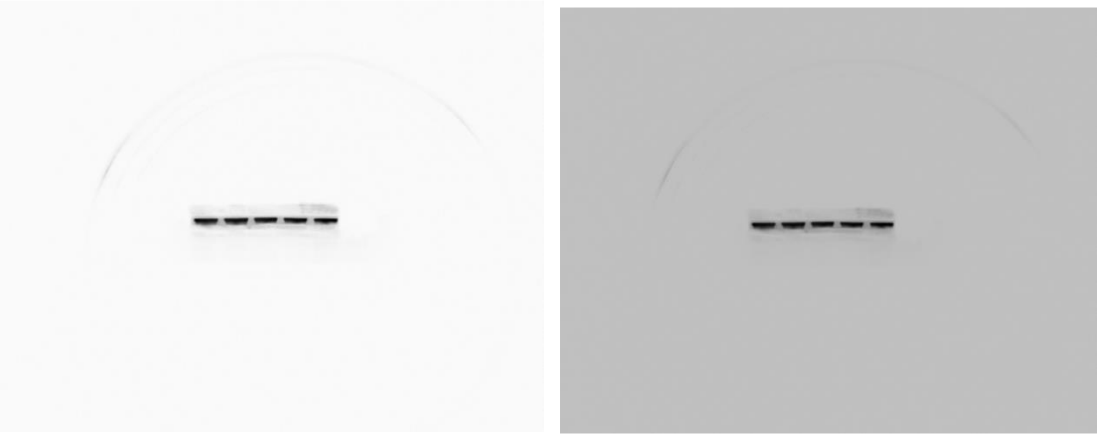

Fig.s3 Cutting original blots/gels for protein expression detection of LC3-II, p62, Beclin-1, p-mTOR, and p-Akt after PI3K inhibitor LY294002 (10 mM) was injected before cornin (10 mg/kg) treatment in CI/R rats. Note: All the SDS-PAGE blots gel cut before the PVDF membrane transfer according to the "Protein Marker", so no full-length blots were provided in this experiment. The gel in the left is high exposure, and the gel in the right is the low exposure for targets protein.

A: p-mTOR

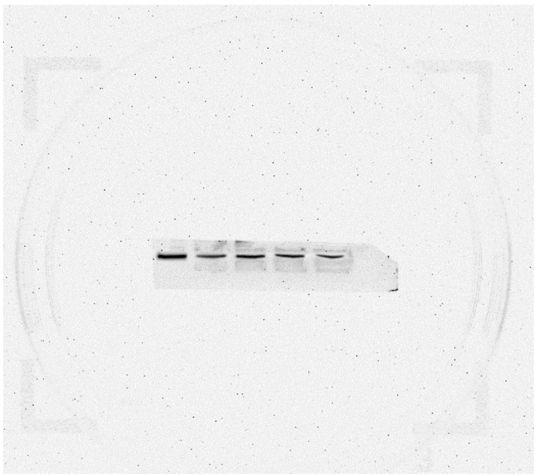

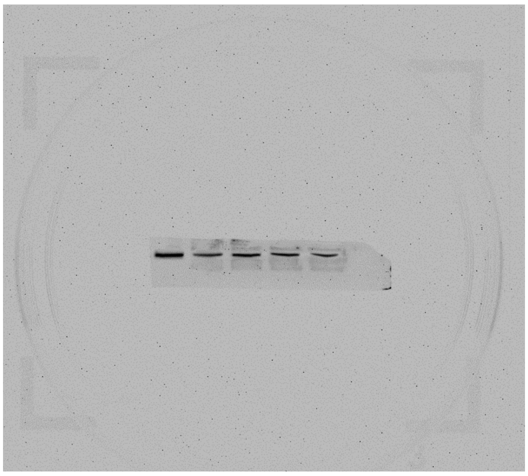

B: mTOR

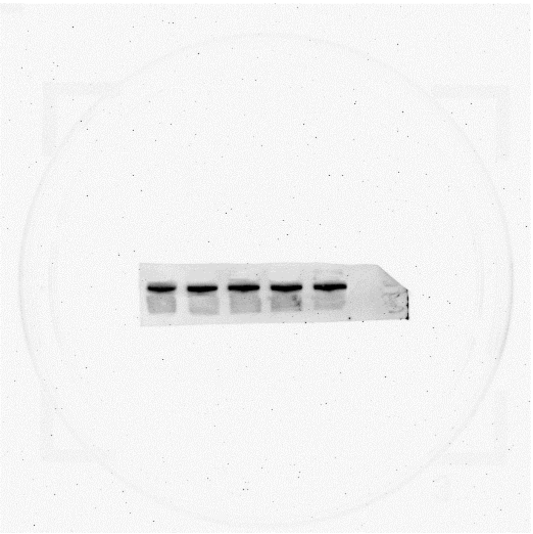

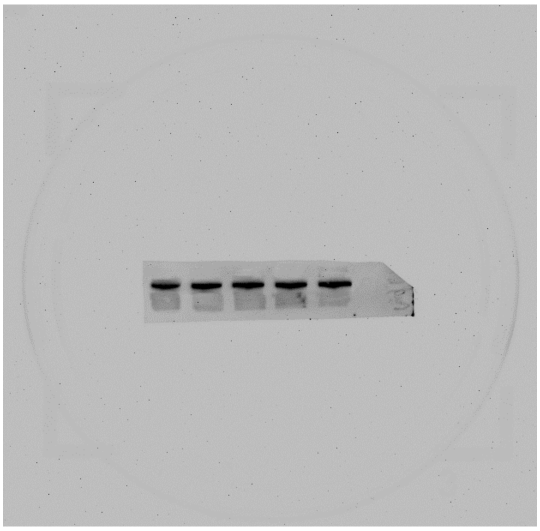

C: p-Akt

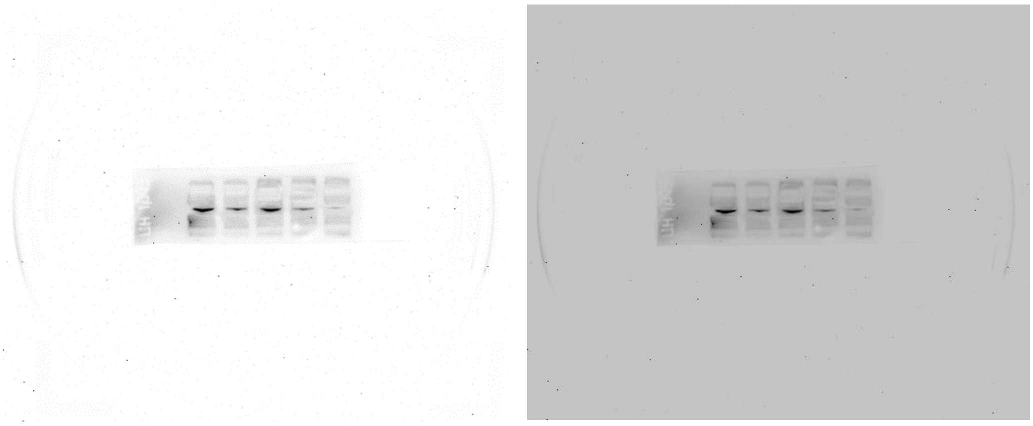

D: Akt

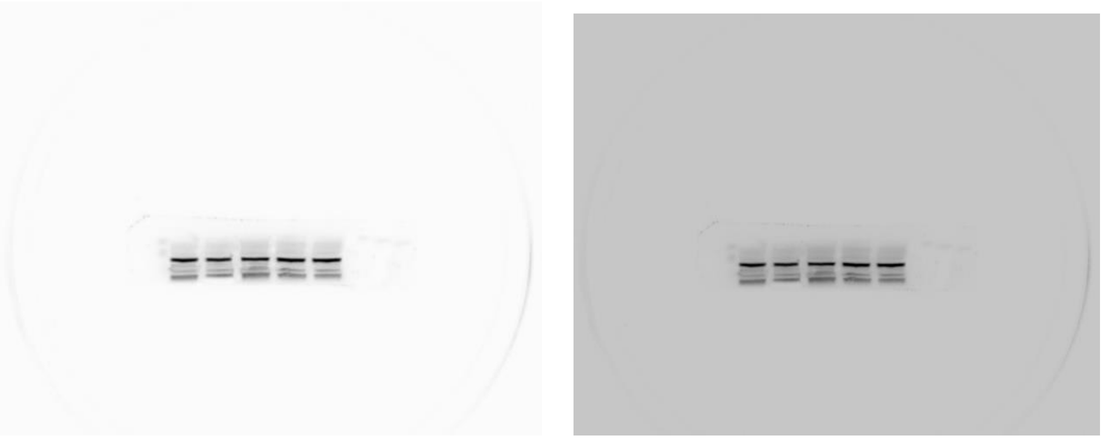

E: p62

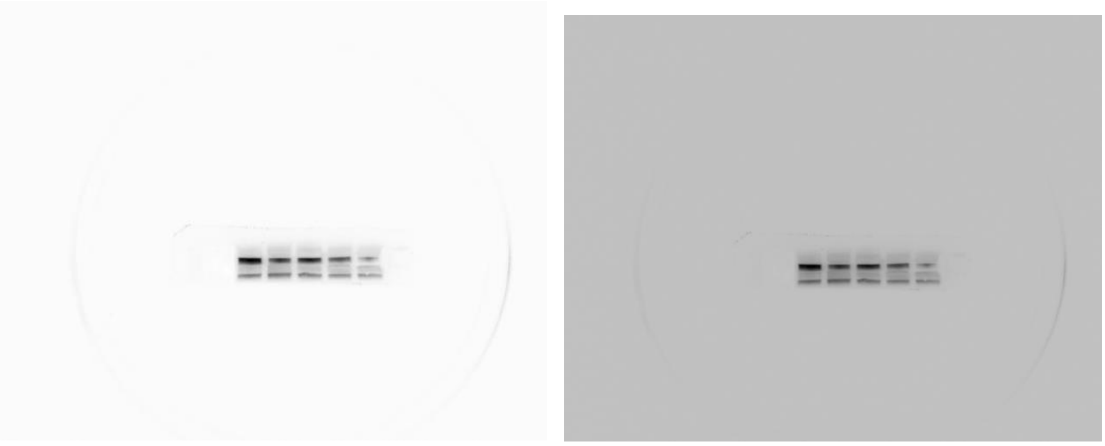

F: Beclin-1

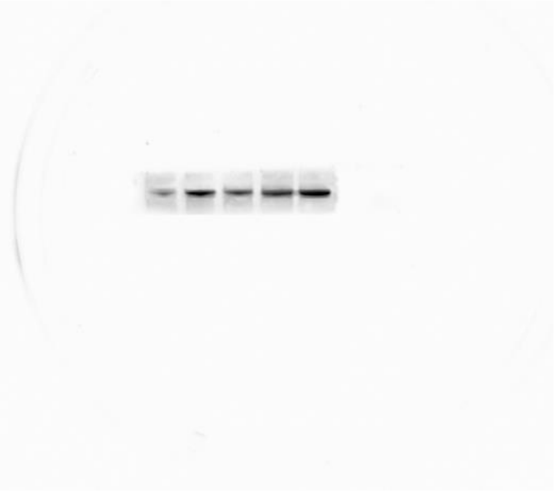

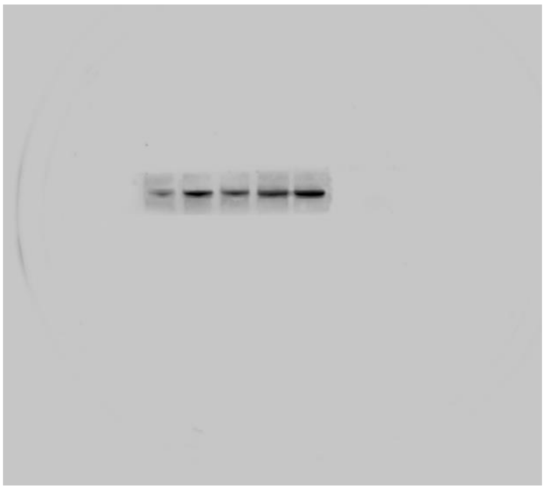

G: LC3-II

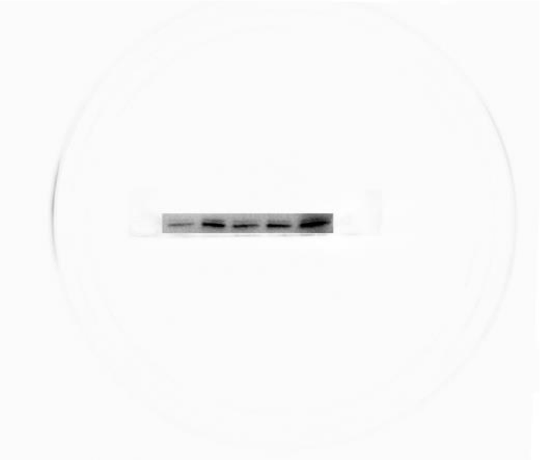

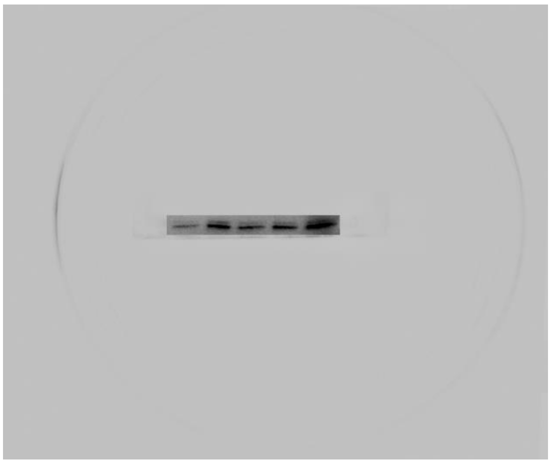

H: GAPDH

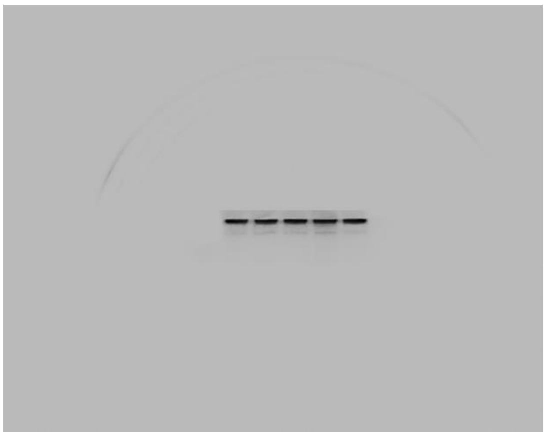

Fig.s4 Cutting original blots/gels for protein expression detection of GFAP, Bax and Bcl-2 after PI3K inhibitor LY294002 (10 mM) was injected before cornin (10 mg/kg) treatment in CI/R rats. Note: All the SDS-PAGE blots gel cut before the PVDF membrane transfer according to the "Protein Marker", so no full-length blots were provided in this experiment. The gel in the left is high exposure, and the gel in the right is the low exposure for targets protein.

A: Bcl-2

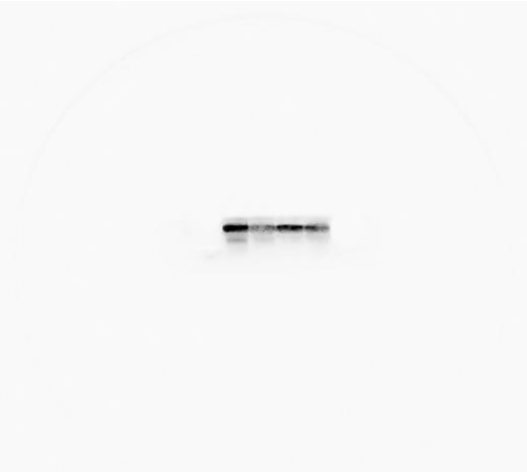

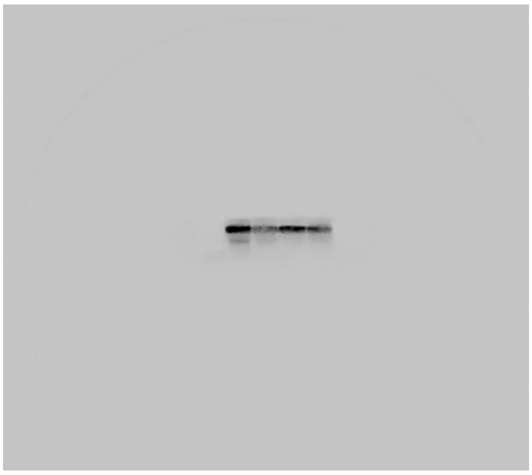

B: Bax

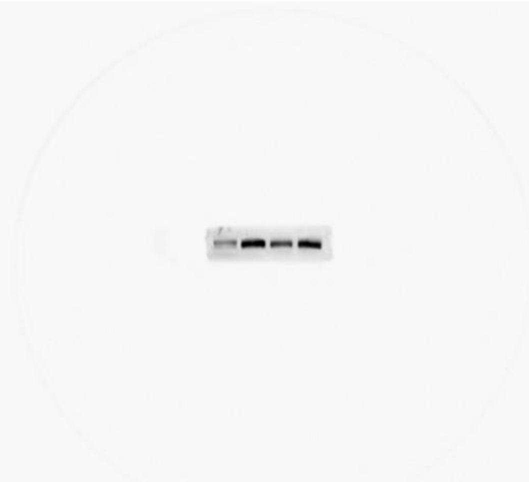

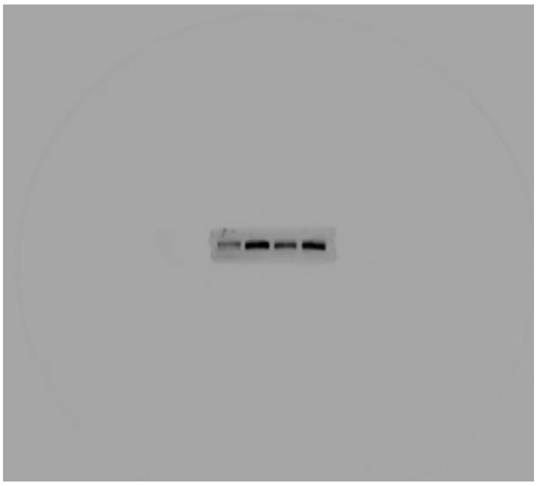

C: GFAP

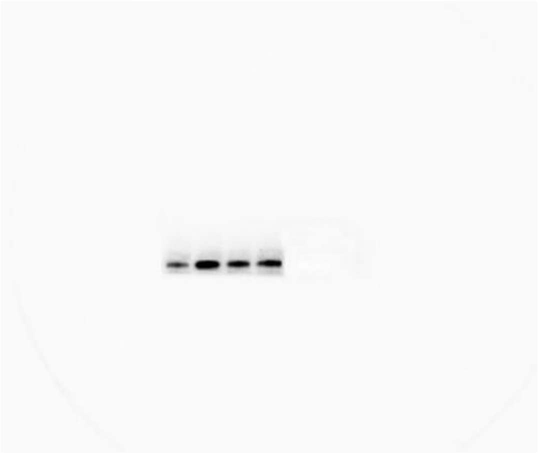

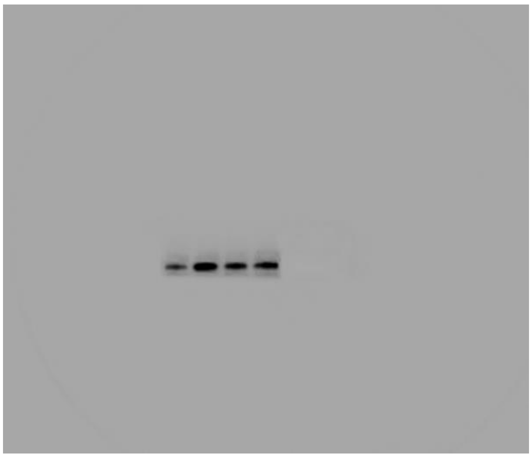

D: GAPDH

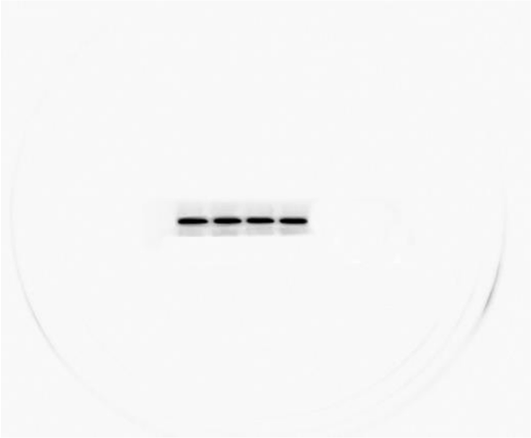

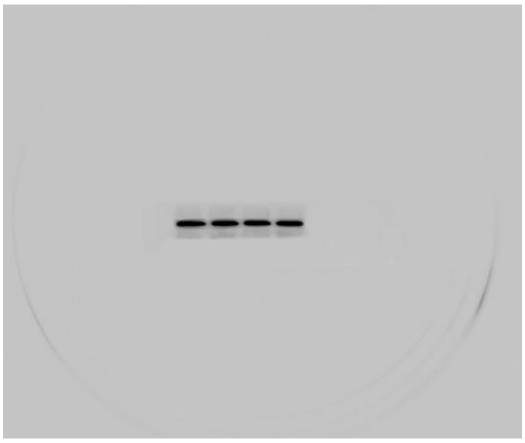

Fig.s5 Cutting original blots/gels for protein expression detection of of LC3-II, p62, Beclin-1, p-mTOR, and p-Akt after PI3K inhibitor LY294002 (10 μM) was used as pretreatment for 6 h, and then incubated with cornin (100 nM) for 48 h in U87 cells. Note: All the SDS-PAGE blots gel cut before the PVDF membrane transfer according to the "Protein Marker", so no full-length blots were provided in this experiment. The gel in the left is high exposure, and the gel in the right is the low exposure for targets protein.

A: p-mTOR

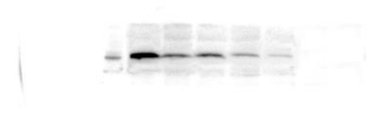

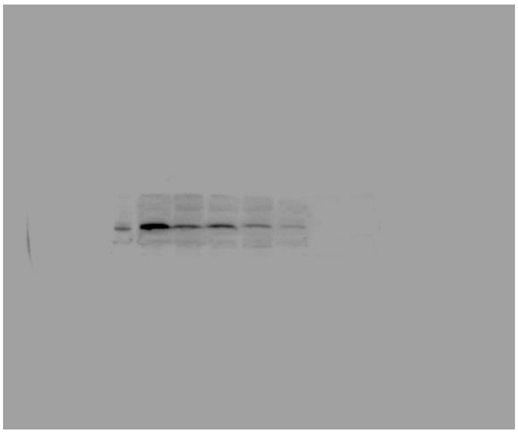

B: mTOR

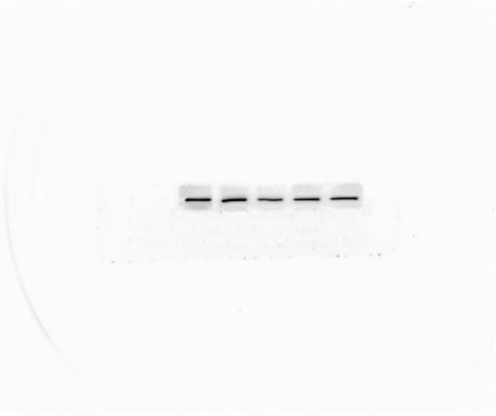

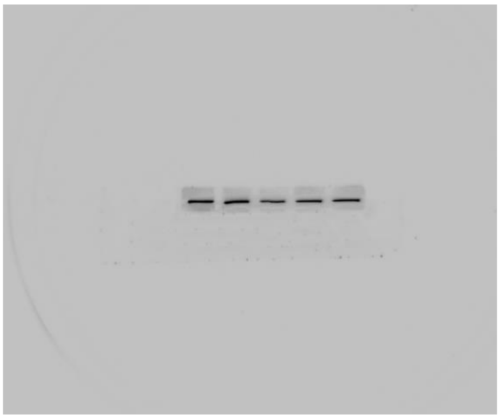

C: p-Akt

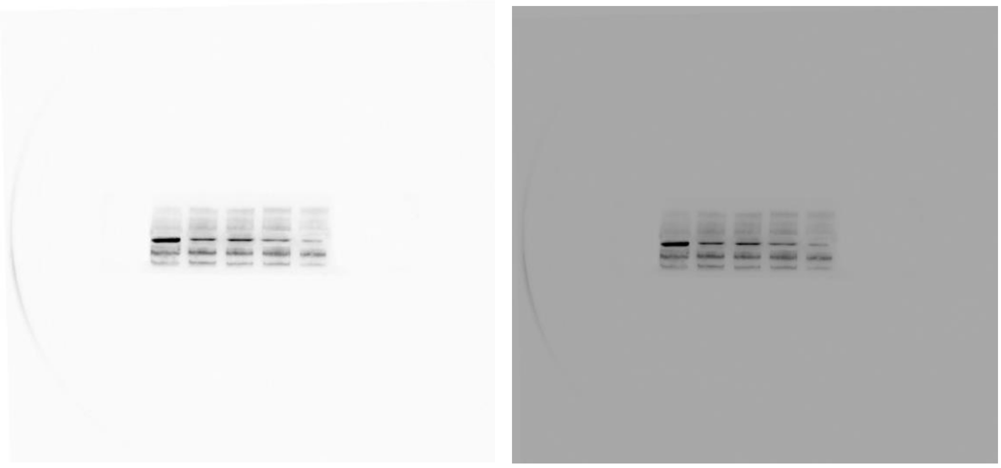

D: Akt

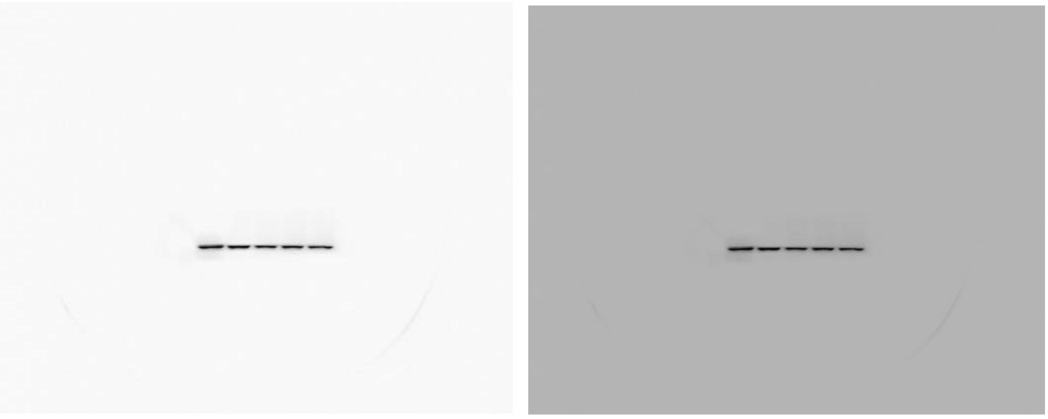

E: p62

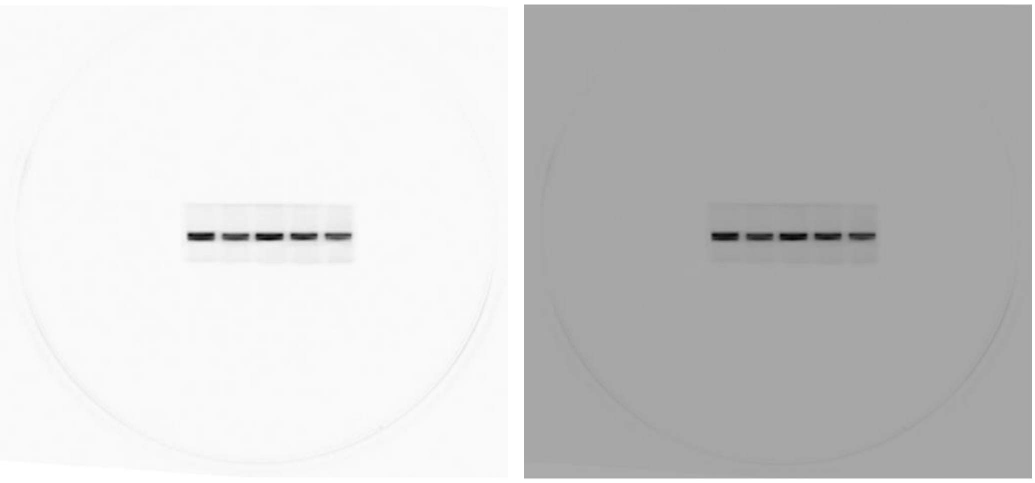

F: Beclin-1

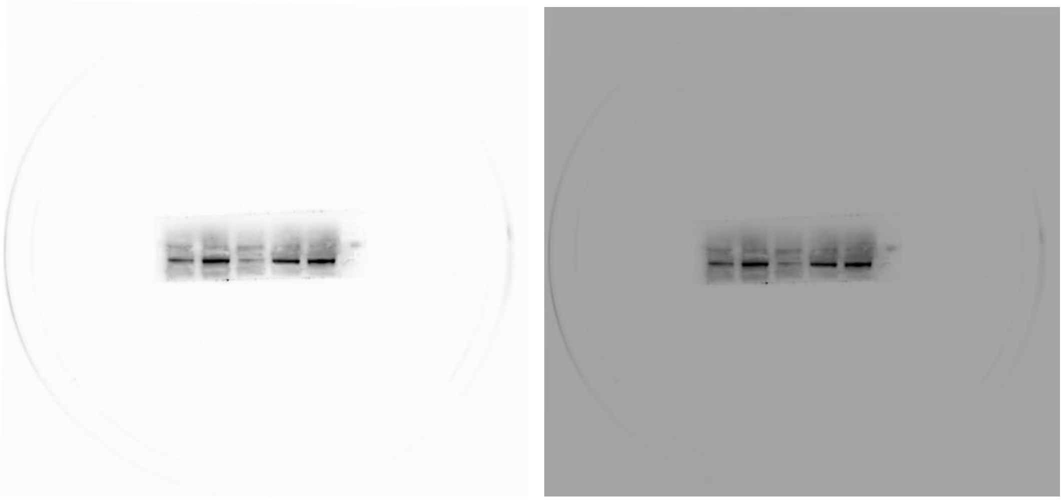

G: LC3-II

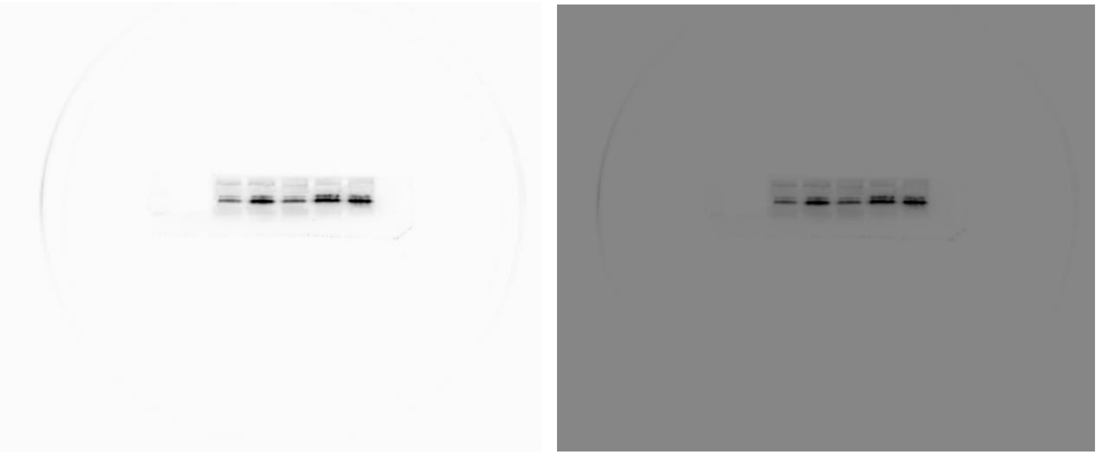

H: β-actin

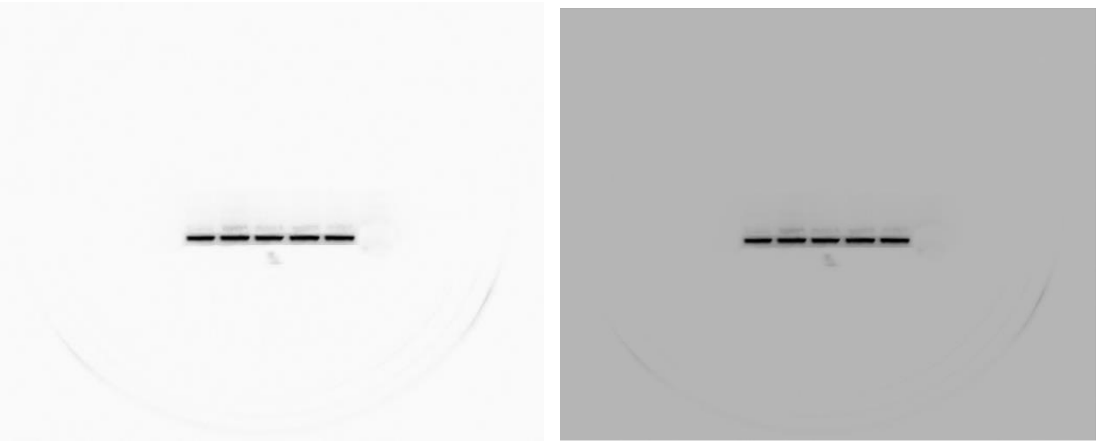

Fig.s6 Cutting original blots/gels for protein expression detection of of p-mTOR, mTOR, LC3-II and p62 after mTOR SiRNA was used as pretreatment for 6 h, and then incubated with cornin (100 nM) for 48 h in U87 cells. Note: All the SDS-PAGE blots gel cut before the PVDF membrane transfer according to the "Protein Marker", so no full-length blots were provided in this experiment. The gel in the left is high exposure, and the gel in the right is the low exposure for targets protein.

A: p-mTOR

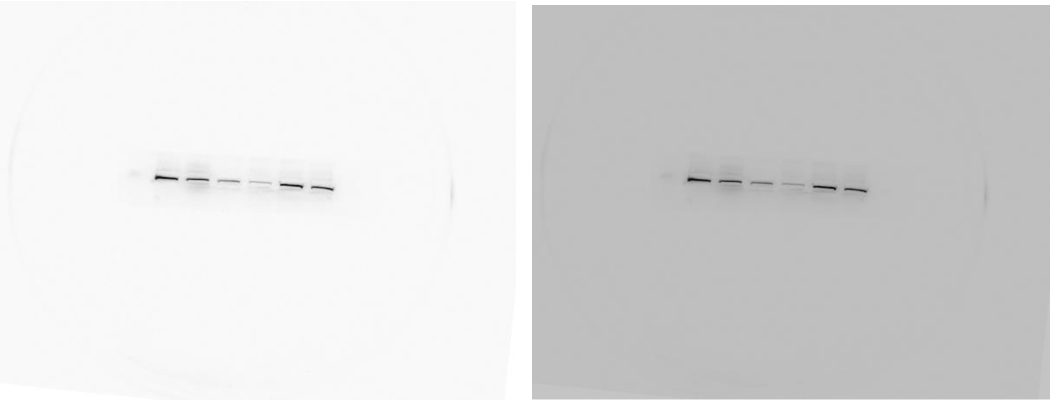

B: mTOR

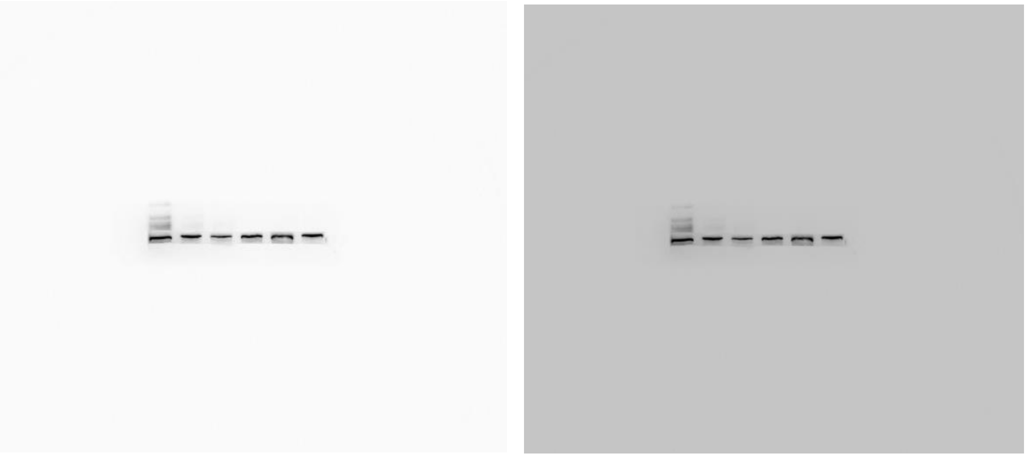

C: p62

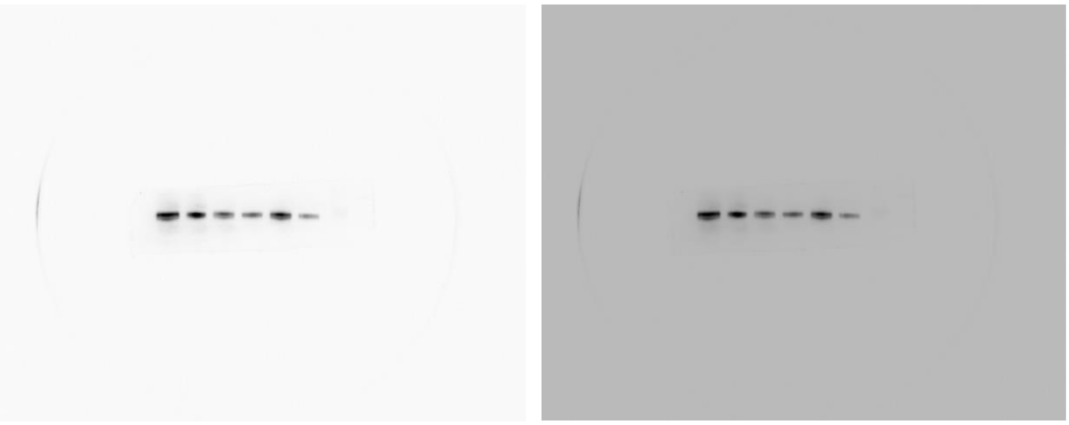

D: LC3-II

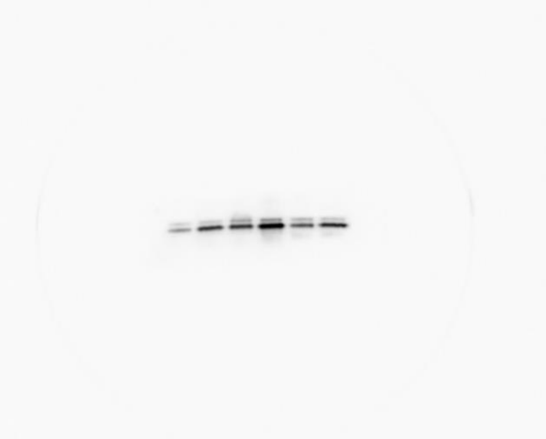

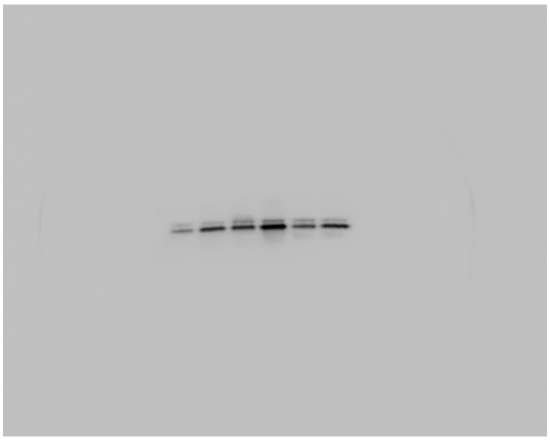

E: β-actin

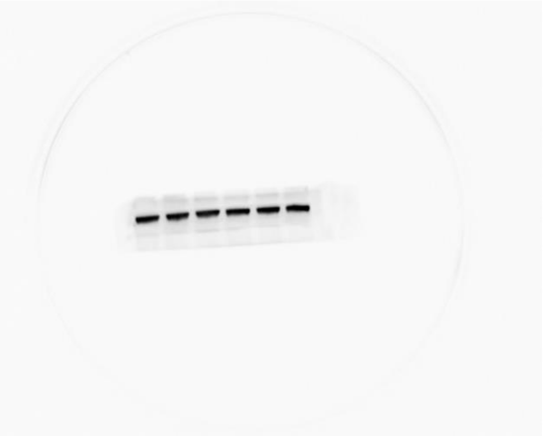

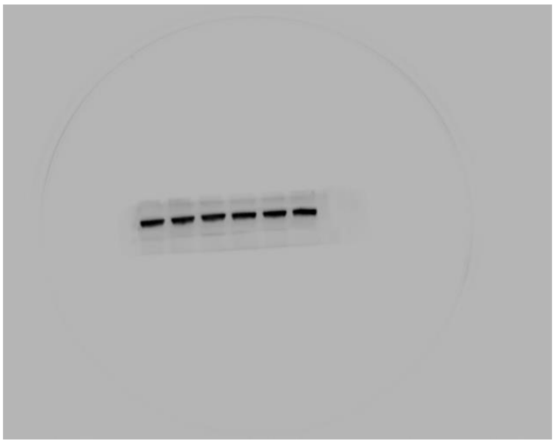

Supplement: Supplementary file 1 — Additional file 1. Supplementary materials. [file 40360_2022_620_MOESM1_ESM.pdf]
